# Proceedings of the 14th annual conference of INEBRIA: New York, NY, USA, September 14–15, 2017

**DOI:** 10.1186/s13722-017-0087-8

**Published:** 2017-09-14

**Authors:** Aisha S. Holloway, Jennifer Ferguson, Sarah Landale, Laura Cariola, Dorothy Newbury-Birch, Amy Flynn, John R. Knight, Lon Sherritt, Sion K. Harris, Amy J. O’Donnell, Eileen Kaner, Barbara Hanratty, Amy M. Loree, Kimberly A. Yonkers, Steven J. Ondersma, Kate Gilstead-Hayden, Steve Martino, Angeline Adam, Robert P. Schwartz, Li-Tzy Wu, Geetha Subramaniam, Gaurav Sharma, Jennifer McNeely, Anne H. Berman, Karoline Kolaas, Elisabeth Petersén, Preben Bendtsen, Erik Hedman, Catharina Linderoth, Ulrika Müssener, Kristina Sinadinovic, Fredrik Spak, Ida Gremyr, Anna Thurang, Ann M. Mitchell, Deborah Finnell, Christine L. Savage, Khadejah F. Mahmoud, Benjamin C. Riordan, Tamlin S. Conner, Jayde A. M. Flett, Damian Scarf, Bonnie McRee, Janice Vendetti, Karen Steinberg Gallucci, Kate Robaina, Brendan J. Clark, Jacqueline Jones, Kathryne D. Reed, Rachel M. Hodapp, Ivor Douglas, Ellen L. Burnham, Laura Aagaard, Paul F. Cook, Brett R. Harris, Jiang Yu, Margaret Wolff, Meighan Rogers, Carolina Barbosa, Brendan J. Wedehase, Laura J. Dunlap, Shannon G. Mitchell, Kristi A. Dusek, Jan Gryczynski, Arethusa S. Kirk, Marla T. Oros, Colleen Hosler, Kevin E. O’Grady, Barry S. Brown, Colin Angus, Sidney Sherborne, Duncan Gillespie, Petra Meier, Alan Brennan, Divane de Vargas, Janaina Soares, Donna Castelblanco, Kelly M. Doran, Ian Wittman, Donna Shelley, John Rotrosen, Lillian Gelberg, E. Jennifer Edelman, Stephen A. Maisto, Nathan B. Hansen, Christopher J. Cutter, Yanhong Deng, James Dziura, Lynn E. Fiellin, Patrick G. O’Connor, Roger Bedimo, Cynthia Gibert, Vincent C. Marconi, David Rimland, Maria C. Rodriguez-Barradas, Michael S. Simberkoff, Amy C. Justice, Kendall J. Bryant, David A. Fiellin, Emma L. Giles, Simon Coulton, Paolo Deluca, Colin Drummond, Denise Howel, Elaine McColl, Ruth McGovern, Stephanie Scott, Elaine Stamp, Harry Sumnall, Luke Vale, Viviana Alabani, Amanda Atkinson, Sadie Boniface, Jo Frankham, Eilish Gilvarry, Nadine Hendrie, Nicola Howe, Grant J. McGeechan, Amy Ramsey, Grant Stanley, Justine Clephane, David Gardiner, John Holmes, Neil Martin, Colin Shevills, Melanie Soutar, Felicia W. Chi, Constance Weisner, Thekla B. Ross, Jennifer Mertens, Stacy A. Sterling, Gillian W. Shorter, Nick Heather, Jeremy Bray, Hildie A. Cohen, Tracy L. McPherson, Cyrille Adam, Hugo López-Pelayo, Antoni Gual, Lidia Segura-Garcia, Joan Colom, India J. Ornelas, Suzanne Doyle, Dennis Donovan, Bonnie Duran, Vanessa Torres, Jacques Gaume, Véronique Grazioli, Cristiana Fortini, Sophie Paroz, Nicolas Bertholet, Jean-Bernard Daeppen, Jason M. Satterfield, Steven Gregorich, Nicholas J. Alvarado, Ricardo Muñoz, Gozel Kulieva, Maya Vijayaraghavan, Angéline Adam, John A. Cunningham, Estela Díaz, Jorge Palacio-Vieira, Alexandra Godinho, Vladyslav Kushir, Kimberly H. M. O’Brien, Laika D. Aguinaldo, Christina M. Sellers, Anthony Spirito, Grace Chang, Tiffany Blake-Lamb, Lea R. Ayers LaFave, Kathleen M. Thies, Amy L. Pepin, Kara E. Sprangers, Martha Bradley, Shasta Jorgensen, Nico A. Catano, Adelaide R. Murray, Deborah Schachter, Ronald M. Andersen, Guillermina Natera Rey, Mani Vahidi, Melvin W. Rico, Sebastian E. Baumeister, Magnus Johansson, Christina Sinadinovic, Ulric Hermansson, Sven Andreasson, Megan A. O’Grady, Sandeep Kapoor, Cherine Akkari, Camila Bernal, Kristen Pappacena, Jeanne Morley, Mark Auerbach, Charles J. Neighbors, Nancy Kwon, Joseph Conigliaro, Jon Morgenstern, Molly Magill, Timothy R. Apodaca, Brian Borsari, Ariel Hoadley, J. Scott Tonigan, Theresa Moyers, Niamh M. Fitzgerald, Lisa Schölin, Nicolas Barticevic, Soledad Zuzulich, Fernando Poblete, Pablo Norambuena, Paul Sacco, Laura Ting, Michele Beaulieu, Paul George Wallace, Matthew Andrews, Kate Daley, Don Shenker, Louise Gallagher, Rod Watson, Tim Weaver, Pol Bruguera, Clara Oliveras, Carolina Gavotti, Pablo Barrio, Fleur Braddick, Laia Miquel, Montse Suárez, Carla Bruguera, Richard L. Brown, Julie Whelan Capell, D. Paul Moberg, Julie Maslowsky, Laura A. Saunders, Ryan P. McCormack, Joy Scheidell, Mirelis Gonzalez, Sabrina Bauroth, Weiwei Liu, Dawn L. Lindsay, Piper Lincoln, Holly Hagle, Sara Wallhed Finn, Anders Hammarberg, Sven Andréasson, Sarah E. King, Rachael Vargo, Brayden N. Kameg, Shauna P. Acquavita, Ruth Anne Van Loon, Rachel Smith, Bonnie J. Brehm, Tiffiny Diers, Karissa Kim, Andrea Barker, Ashley L. Jones, Asheley C. Skinner, Agatha Hinman, Dace S. Svikis, Casey L. Thacker, Ken Resnicow, Jessica R. Beatty, James Janisse, Karoline Puder, Ann-Sofie Bakshi, Joanna M. Milward, Andreas Kimergard, Claire V. Garnett, David Crane, Jamie Brown, Robert West, Susan Michie, Ingvar Rosendahl, Claes Andersson, Mikael Gajecki, Matthijs Blankers, Kim Donoghue, Ellen Lynch, Ian Maconochie, Ceri Phillips, Rhys Pockett, Tom Phillips, R. Patton, Ian Russell, John Strang, Maureen T. Stewart, Amity E. Quinn, Mary Brolin, Brooke Evans, Constance M. Horgan, Junqing Liu, Fern McCree, Doug Kanovsky, Tyler Oberlander, Huan Zhang, Ben Hamlin, Robert Saunders, Mary B. Barton, Sarah H. Scholle, Patricia Santora, Chirag Bhatt, Kazi Ahmed, Dominic Hodgkin, Wenwu Gao, Elizabeth L. Merrick, Charles E. Drebing, Mary Jo Larson, Monica Sharma, Nancy M. Petry, Richard Saitz, Constance M. Weisner, Kelly C. Young-Wolff, Wendy Y. Lu, John R. Blosnich, Keren Lehavot, Joseph E. Glass, Emily C. Williams, Kara M. Bensley, Gary Chan, Julie Dombrowski, John Fortney, Anna D. Rubinsky, Gwen T. Lapham, Ariadna Forray, Todd A. Olmstead, Kathryn Gilstad-Hayden, Trace Kershaw, Pamela Dillon, Michael F. Weaver, Emily R. Grekin, Jennifer D. Ellis, Lucy McGoron, Lucy McGoron

**Affiliations:** 1 Nursing Studies, School of Health in Social Science, The University of Edinburgh, Edinburgh, UK; 2 Health and Social Care Institute, School of Health and Social Care, Teesside University, Middlesbrough, Tees Valley UK; 3 Department of Pediatrics, Harvard Medical School, Boston, MA USA; 4 Center for Adolescent Substance Abuse Research, Boston Children’s Hospital, Boston, MA USA; 5 Institute of Health and Society, Newcastle University, Baddiley-Clark Building, Richardson Road, Newcastle Upon Tyne, Northumberland UK; 6 VA Connecticut Healthcare System, Veterans Aging Cohort Study, West Haven, CT USA; 7 Department of Psychiatry, Yale University School of Medicine, New Haven, CT USA; 8 Merrill-Palmer Skillman Institute and Department of Psychiatry and Behavioral Neurosciences, Wayne State University, Detroit, MI USA; 9 Department of Population Health, NYU School of Medicine, New York, NY USA; 10 Friends Research Institute, Inc, Baltimore, MD USA; 11 Department of Psychiatry and Behavioral Sciences, Department of Medicine and Duke Clinical Research Institute, Duke University Medical Center, Durham, NC USA; 12 Center for the Clinical Trials Network, National Institute on Drug Abuse, National Institutes of Health, Bethesda, MD USA; 13 The EMMES Corporation, Rockville, MD USA; 14 Department of Clinical Neuroscience, Center for Psychiatry Research, Karolinska Institutet, Stockholm, Sweden; 15 Gustavsberg Primary Care Clinic, Odelbergs väg 19, Gustavsberg, Sweden; 16 Stockholm Center for Dependency Disorders, Box 17914, Stockholm, Sweden; 17 Department of Medicine and Health Sciences, Linköping University, Linköping, Sweden; 18 Department of Social Medicine, Gothenburg University, Gothenburg, Sweden; 19 Division of Service Management and Logistics, Chalmers Technological University, Göteborg, Sweden; 20 Department of Health and Community Systems, University of Pittsburgh School of Nursing, Pittsburgh, PA USA; 21 Department of Acute and Chronic Care, Johns Hopkins University, Baltimore, MD USA; 22 School of Nursing, Johns Hopkins University, Baltimore, MD USA; 23 School of Nursing, University of Pittsburgh, Pittsburgh, PA USA; 24 Department of Psychology, University of Otago, Dunedin, New Zealand; 25 Department of Community Medicine and Health Care, University of Connecticut School of Medicine, Farmington, CT USA; 26 Division of Pulmonary Sciences and Critical Care Medicine, School of Medicine, University of Colorado, Aurora, CO USA; 27 College of Nursing, University of Colorado, Aurora, CO USA; 28 Department of Health Policy, Management, and Behavior, University at Albany School of Public Health, Rensselaer, NY USA; 29 Center on Addictions Research, University at Albany School of Social Welfare, Albany, NY USA; 30 Mount Sinai Icahn School of Medicine, New York, NY USA; 31 Bureau of STD Control, NYC Department of Health and Mental Hygiene, Long Island City, USA; 32 Behavioral Health Economics Program, RTI International, Chicago, IL USA; 33 Behavioral Health Economics Program, RTI International, Research Triangle Park, NC USA; 34 Social Research Center, Friends Research Institute, Baltimore, MD USA; 35 Total Health Care, Baltimore, MD USA; 36 Mosaic Group, Towson, MD USA; 37 Department of Psychology, University of Maryland, College Park, MD USA; 38 Department of Psychology, University of North Carolina Wilmington, Wilmington, NC USA; 39 School of Health and Related Research, University of Sheffield, Sheffield, UK; 40 Department of Maternal-Infant and Psychiatric Nursing, University of São Paulo School of Nursing, São Paulo, Brazil; 41 Department of Emergency Medicine, NYU School of Medicine, New York, NY USA; 42 Departments of Emergency Medicine and Population Health, NYU School of Medicine, New York, NY USA; 43 Departments of Medicine and Population Health, NYU School of Medicine, New York, NY USA; 44 Department of Psychiatry, NYU School of Medicine, New York, NY USA; 45 Department of Family Medicine, David Geffen School of Medicine at UCLA; Department of Health Policy and Management, UCLA Fielding School of Public Health; Office of Healthcare Transformation and Innovation, VA Greater Los Angeles Healthcare System, Los Angeles, CA USA; 46 Yale University School of Medicine, New Haven, CT USA; 47 Center for Interdisciplinary Research on AIDS, Yale University School of Public Health, New Haven, CT USA; 48 Syracuse University, Syracuse, NY USA; 49 College of Public Health, University of Georgia, Athens, GA USA; 50 Yale Center for Analytic Sciences, Yale University School of Public Health, New Haven, CT USA; 51 Veterans Affairs North Texas Health Care System and UT Southwestern, Dallas, TX USA; 52 D.C. Veterans Affairs Medical Center, George Washington University School of Medicine and Health Sciences, Washington, DC USA; 53 Emory University School of Medicine, Atlanta Veterans Affairs Medical Center, Decatur, GA USA; 54 Michael E. DeBakey Veterans Affairs Medical Center, Baylor College of Medicine, Houston, TX USA; 55 VA NY Harbor Healthcare System and New York University School of Medicine, New York, NY USA; 56 National Institute on Alcohol Abuse and Alcoholism HIV/AIDS Program, Bethesda, MD USA; 57 School of Health and Social Care, Teesside University, Middlesbrough, Tees Valley, UK; 58 Centre for Health Services Studies, University of Kent, Canterbury, Kent, UK; 59 Addictions Department, Institute of Psychiatry, Psychology and Neuroscience, King’s College London, London, UK; 60 School of Education, Communication and Language Sciences, Newcastle University, Newcastle Upon Tyne, UK; 61 Health Economics Group, Institute of Health and Society, Newcastle University, Richardson Road, Newcastle Upon Tyne, UK; 62 Centre for Public Health, Liverpool John Moores University, Liverpool, UK; 63 Faculty of Education, Health and Community, Liverpool John Moores University, Liverpool, UK; 64 Northumberland, Tyne and Wear NHS Foundation Trust, Newcastle Upon Tyne, UK; 65 Newcastle Clinical Trials Unit, Newcastle University, Newcastle Upon Tyne, UK; 66 Education Service, Wellbeing and Community Health Services Group, Brunel Building, 64 Regent Street, Blyth, UK; 67 Public Health England North East, Waterfront 4, Goldcrest Way, Newburn Riverside, Newcastle Upon Tyne, Tyne and Wear UK; 68 Section of Public Health ScHARR, University of Sheffield, Regent Court, 30 Regent Street, Sheffield, UK; 69 Balance North East, Bede House, Durham, UK; 70 Matrix Young People’s Service, 7 Burrow Street, South Shields, Tyne and Wear UK; 71 Division of Research, Kaiser Permanente Northern California, Oakland, CA USA; 72 Department of Psychiatry, University of California, San Francisco, San Francisco, CA USA; 73 Aurora Public Schools Division of Accountability and Research, Educational Services Center 1, Aurora, CO USA; 74 Alcohol and Public Health Team, School of Health and Social Care, Middlesbrough, Teesside UK; 75 Faculty of Health and Life Sciences, Northumbria University, Newcastle, Northumberland UK; 76 Bryan School of Business and Economics, University of North Carolina at Greensboro, Greensboro, NC USA; 77 NORC at the University of Chicago, Chicago, IL USA; 78 Public Health, NORC at the University of Chicago, Bethesda, MD USA; 79 Research and Development, Kognito, New York, NY USA; 80 Grup de Recerca Addicions Clínic, Hospital Clínic de Barcelona, IDIBAPS, University of Barcelona, Barcelona, Catalonia Spain; 81 Program on Substance Abuse, Public Health Agency, Government of Catalonia, Barcelona, Catalonia Spain; 82 Department of Health Services, University of Washington, Seattle, WA USA; 83 Alcohol and Drug Abuse Institute, University of Washington, Seattle, WA USA; 84 School of Social Work, University of Washington, Seattle, WA USA; 85 Alcohol Treatment Center, Lausanne University Hospital, Lausanne, Switzerland; 86 Department of General Internal Medicine, University of California San Francisco, San Francisco, CA USA; 87 PhD Program in Clinical Psychology, Palo Alto University, Palo Alto, CA USA; 88 Department of Medicine, University of California San Francisco, San Francisco, CA USA; 89 Department of Community Medicine, Lausanne University, Lausanne, Switzerland; 90 Centre for Addiction and Mental Health, Toronto, ON Canada; 91 Addiction Unit, Hospital Clínic, Barcelona, Spain; 92 Department of Health and Human Development, Education Development Center, Waltham, MA USA; 93 Department of Social Work, The Ethelyn R. Strong School of Social Work at Norfolk State University, Norfolk, VA USA; 94 Department of Social Work, Boston College School of Social Work, Chestnut Hill, MA USA; 95 Department of Psychiatry and Human Behavior, Brown University, Providence, RI USA; 96 Department of Psychiatry, Yale School of Medicine, VA Connecticut Healthcare, New Haven, CT USA; 97 Department of Psychiatry and Behavioral Neurosciences, Merrill-Palmer Skillman Institute, Detroit, MI USA; 98 Department of Psychiatry, VA Boston Healthcare and Harvard University, Boston, MA USA; 99 Department of Obstetrics and Gynecology, Massachusetts General Hospital, Boston, MA USA; 100 JSI Research and Training Institute, Inc, Bow, NH USA; 101 TLQ Associates Healthcare Consulting LLC, Bedford, NH USA; 102 New Hampshire Charitable Foundation, Concord, NH USA; 103 Department of Family Medicine, David Geffen School of Medicine at UCLA, Los Angeles, CA USA; 104 Fielding School of Public Health, University of California, Los Angeles, Los Angeles, CA USA; 105 National Institute of Psychiatry Ramón de la Fuente Muñiz, Mexico City, Mexico; 106 Department of Epidemiology and Preventive Medicine, University of Regensburg, Regensburg, Germany; 107 Department of Public Health Sciences, Karolinska Institutet, Stockholm, Sweden; 108 Department of Clinical Neuroscience, Karolinska Institutet, Stockholm, Sweden; 109 Division of Health Services Research, The National Center on Addiction and Substance Abuse, New York, NY USA; 110 Department of Internal Medicine, Emergency Medicine, and Psychiatry, Northwell Health, Great Neck, NY USA; 111 Department of Emergency Medicine, Northwell Health, Great Neck, NY USA; 112 Department of Psychiatry, Northwell Health, Great Neck, NY USA; 113 Center for Alcohol and Addiction Studies, Brown University, Providence, RI USA; 114 Children’s Mercy Kansas City, University of Missouri – Kansas City School of Medicine, Kansas City, MO USA; 115 San Francisco Veterans Affairs Health System and Department of Psychiatry, University of San Francisco, San Francisco, CA USA; 116 School of Public Health, Brown University, Providence, RI USA; 117 Center on Alcoholism, Substance Abuse, and Addictions, University of New Mexico, Albuquerque, NM USA; 118 Institute for Social Marketing, UK Centre for Tobacco and Alcohol Studies, University of Stirling, Stirling, Scotland, UK; 119 World Health Organization Regional Office for Europe, formerly Institute for Social Marketing, University of Stirling, Stirling, Scotland, UK; 120 Alcohol Treatment Center, Department of Community Medicine and Health, Lausanne University Hospital, Lausanne, Switzerland; 121 Department of Family Medicine, Pontificia Universidad Católica de Chile, Santiago de Chile, Región Metropolitana Chile; 122 Nursing School, Pontificia Universidad Católica de Chile, Santiago de Chile, Región Metropolitana Chile; 123 Public Health Department, Pontificia Universidad Católica de Chile, Santiago de Chile, Región Metropolitana Chile; 124 Mental Health Department, Ministry of Health, Santiago de Chile, Región Metropolitana Chile; 125 School of Social Work, University of Maryland-Baltimore, Baltimore, MD USA; 126 University of Maryland-Baltimore County, Baltimore, MD USA; 127 South London Health Innovation Network, London, UK; 128 Safe Sociable London Partnership, London, UK; 129 Alcohol Health Network, London, UK; 130 Department of Public Health, Royal Borough of Kingston, London, UK; 131 Middlesex University, London, UK; 132 Grup de Recerca Addiccions Clínic (GRAC), Addictive Behaviours Unit, Psychiatry Department, Hospital Clínic de Barcelona, Barcelona, Spain; 133 Psychiatry Department, Hospital Clínic de Barcelona, Barcelona, Spain; 134 Emergency Department, Hospital Clínic de Barcelona, Barcelona, Spain; 135 Program on Substance Use, Public Health Agency, Government of Catalonia, Barcelona, Spain; 136 Department of Family Medicine and Community Health, University of Wisconsin School of Medicine and Public Health, Madison, WI USA; 137 Julie INK, LLC, Milwaukee, WI USA; 138 Population Health Institute, University of Wisconsin-Madison, Madison, WI USA; 139 Department of Kinesiology and Health Education, University of Texas-Austin, Austin, TX USA; 140 Research and Evaluation, Institute for Research, Education and Training in Addiction, Pittsburgh, PA USA; 141 Education and Training, Education and Training, Institute for Research in Addiction, Pittsburgh, PA USA; 142 Department of Public Health Sciences, Karolinska Institutet, Centre for Psychiatry Research, Stockholm Health Care Services, Stockholm, Sweden; 143 Public Health Department, NORC at the University of Chicago, Bethesda, MD USA; 144 Training and Education, Institute for Research, Education and Training in Addiction, Pittsburgh, PA USA; 145 School of Social Work, College of Allied Health Sciences, University of Cincinnati, Cincinnati, OH USA; 146 School of Social Work, University of Cincinnati, Cincinnati, OH USA; 147 College of Nursing, University of Cincinnati, Cincinnati, OH USA; 148 College of Medicine, University of Cincinnati, Cincinnati, OH USA; 149 James L. Winkle College of Pharmacy, University of Cincinnati, Cincinnati, OH USA; 150 Department of Medicine, Clinical Research Institute, Duke University, Durham, NC USA; 151 Department of Psychiatry and Behavioral Neurosciences and Merrill-Palmer Skillman Institute, Wayne State University, Detroit, MI USA; 152 VCU Institute for Women’s Health and Departments of Psychology, Psychiatry and Obstetrics/Gynecology, Virginia Commonwealth University, Richmond, VA USA; 153 School of Public Health, University of Michigan, Ann Arbor, MI USA; 154 Merrill-Palmer Skillman Institute, Wayne State University, Detroit, MI USA; 155 Department of Family Medicine and Public Health Sciences, Wayne State University, Detroit, MI USA; 156 Department of Obstetrics and Gynecology, Wayne State University, Detroit, MI USA; 157 Addictions Department, King’s College London, London, UK; 158 Department of Clinical, Educational and Health Psychology, University College London, London, England; 159 Department of Behavioural Science and Health, University College London, London, England; 160 Department of Criminology, Malmö University, Malmö, Sweden; 161 Trimbos Institute—The Netherlands Institute of Mental Health and Addiction, Utrecht, The Netherlands; 162 Arkin Mental Health Care, Amsterdam, The Netherlands; 163 Department of Psychiatry, Academic Medical Centre, University of Amsterdam, Amsterdam, The Netherlands; 164 Department of Addictions, Institute of Psychiatry, Psychology and Neuroscience, King’s College London, London, UK; 165 Paediatric Emergency Medicine, Imperial College London, London, UK; 166 Swansea Centre for Health Economics, College of Human and Health Sciences, Swansea University, Swansea, Wales, UK; 167 School of Psychology, University of Surrey, Guildford, UK; 168 Swansea University Medical School, Swansea, Wales UK; 169 Institute for Behavioral Health, The Heller School for Social Policy and Management, Brandeis University, Waltham, MA USA; 170 Department of Community Health Sciences, Cumming School of Medicine, University of Calgary, Calgary, AB Canada; 171 National Committee for Quality Assurance, Washington, DC USA; 172 The Substance Abuse and Mental Health Services Administration, Rockville, MD USA; 173 FEi systems, Columbia, MD USA; 174 Institute for Behavioral Health, Schneider Institutes for Health Policy, Heller School for Social Policy and Management, Brandeis University, Waltham, MA USA; 175 Psychology Service, Department of Veterans Affairs Medical Center, Bedford, MA USA; 176 Primary Care Service, Department of Veterans Affairs Medical Center, Bedford, MA USA; 177 University of Connecticut School of Medicine, Farmington, CT USA; 178 Department of Community Health Sciences, Boston University School of Public Health, Boston, MA USA; 179 Division of Research, Kaiser Permanente, Oakland, CA USA; 180 Department of Veterans Affairs, VA Pittsburgh Healthcare System, Center for Health Equity Research and Promotion, Pittsburgh, PA USA; 181 Denver-Seattle Center of Innovation for Veteran-Centered Value-Driven Care, VA Puget Sound Healthcare System, Seattle, WA USA; 182 Kaiser Permanente Washington Health Research Institute, Seattle, WA USA; 183 Department of Health Services, University of Washington School of Public Health, Seattle, WA USA; 184 Department of Health Services and Department of Biostatistics, University of Washington, Seattle, WA USA; 185 Department of Medicine and Allergy and Infectious Diseases, University of Washington, Seattle, WA USA; 186 Department of Psychiatry and Behavioral Sciences, University of Washington, Seattle, WA USA; 187 Kidney Health and Research Collaborative, University of California San Francisco, San Francisco, CA USA; 188 Health Services Research and Development, Veterans Health Administration Puget Sound, Seattle, WA USA; 189 Lyndon B. Johnson School of Public Affairs, University of Texas at Austin, Austin, TX USA; 190 School of Epidemiology and Public Health, Yale School of Medicine, New Haven, CT USA; 191 Department of Psychiatry, Yale School of Medicine, VA Connecticut Healthcare System, West Haven, CT USA; 192 Department of Psychology, Institute for Women’s Health, Virginia Commonwealth University, Richmond, VA USA; 193 Center for Clinical and Translational Research, Virginia Commonwealth University, Richmond, VA USA; 194 University of Texas Health Science Center, Houston, TX USA; 195 Department of Psychology, Wayne State University, Detroit, MI USA

## A1 What does an ABI look like when delivered in the prison setting: perspectives and experiences of male remand prisoners and relevant stakeholders

### Aisha S. Holloway^1^, Jennifer Ferguson^2^, Sarah Landale^3^, Laura Cariola^3^, Dorothy Newbury-Birch^2^

#### ^1^Nursing Studies, School of Health in Social Science, The University of Edinburgh, Edinburgh, UK; ^2^Health and Social Care Institute, School of Health and Social Care, Teesside University, Middlesbrough, Tees Valley, UK; ^3^School of Health in Social Science, The University of Edinburgh, Edinburgh, UK

##### **Correspondence:** Aisha Holloway - aisha.holloway@ed.ac.uk

*Addiction Science & Clinical Practice* 2017, **12(Suppl 1)**: A1

**Background:** Addressing alcohol harm in prisons can potentially reduce the risk of re-offending, and costs to society, whilst tackling health inequalities. Health savings of £4.3 m and crime savings of £100 m per year can be a result of appropriate alcohol interventions. Prison therefore offers an opportunity for the identification, response and/or referral to treatment for those male remand prisoners who are consuming alcohol above recommended levels. There is however, limited evidence for the effectiveness, optimum timing of delivery, recommended length, content, implementation and economic benefit of Alcohol Brief Interventions (ABI) in the prison setting for male remand prisoners. As part of the PRISM-A study, we aimed to explore the ‘elements’ of an acceptable ABI for delivery, experiences of engagement with services/health professionals about alcohol use, alongside barriers and facilitators to implementation within the prison setting for male remand prisoners.

**Materials and methods:** Twenty-four in-depth interviews were conducted with adult male remand prisoners at one Scottish prison (n = 12) and one English prison (n = 12). A focus group at each of the prison sites was held with key stakeholders (e.g. prison nurses, prison officers, voluntary alcohol/addiction services, health service managers and commissioners). Thematic analysis techniques utilizing NViVo 10 were employed.

**Results:** A thematic content analysis of the interviews consistently highlighted that the majority of prisoners reflected about the connection between alcohol consumption and criminal offending, particularly in relation to offenses involving physical assaults. They also expressed motivation to change their alcohol consumption. Both prisoner interviews and focus groups with stakeholders (N = 2), indicated the value of continuous follow-up support outside of the prison system and also the need to address the lack of stable social environments, which is often associated with alcohol and drug consumption. Stakeholders further identified organizational barriers to the delivery of ABI, such as limited funding and manageable workloads.

**Conclusions:** The importance of interpersonal trust indicated that intervention delivery by external organizations and nurses were favored in comparison to intervention delivery by prison staff and peer-prisoners.

## A2 Does perceived risk of harm mediate the effects of a primary care alcohol screening and brief advice intervention for adolescents?

### Amy Flynn, John R. Knight, Lon Sherritt, Sion K. Harris

#### Department of Pediatrics, Harvard Medical School, Boston, MA, USA; Center for Adolescent Substance Abuse Research, Boston Children’s Hospital, Boston, MA, USA

##### **Correspondence:** Amy Flynn - amy.flynn@childrens.harvard.edu

*Addiction Science & Clinical Practice* 2017, **12(Suppl 1)**: A2

**Background:** A previous study found adolescents receiving brief computer-facilitated screening and clinician advice (cSBA) in primary care reported lower rates of alcohol use at follow-up compared to usual care. One intervention component was provision of science-based information about alcohol risks for adolescents’ developing brain and health. A hypothesized intervention mechanism was enhancing perceived risk of harm of use, and this study examined whether perceived risk mediated the intervention effect.

**Materials and methods:** We analyzed data from a quasi-experimental trial of cSBA among 2096 12–18 year-old patients recruited from 9 New England practices. The study used a before-after design with practices being their own control. An 18-month Treatment as Usual (TAU) phase was followed by clinician training and an 18-month cSBA phase with computer-administered screening, individualized feedback, health risk information, and clinician brief advice. We stratified analyses by baseline past-12-month alcohol use and used mediated logistic regression modeling to examine any past-3-month drinking at 3-months. We tested 2 mediator variables representing trajectories from baseline to 3-months in perceived risk of harm (PROH) of trying alcohol, and of binge drinking on weekends. Each trajectory variable had three categories: 2 = stayed high (moderate/great risk), 1 = increased to moderate/great risk, or 0 = stayed at, or declined to, no/low risk. We examined mediation by PROH of trying alcohol among baseline non-users, and PROH of binge drinking among baseline users.

**Results:** Among baseline non-users (n = 1449), the cSBA effect was partially mediated by PROH (trying alcohol) (total effect beta [95% CI] = −0.773 [−1.481, −0.064]; indirect effect −0.066 [−0.206, −0.007]), with cSBA associated with higher PROH over time compared to TAU (0.140 [0.026, 0.255]), and higher PROH decreasing odds of reporting past-3-month drinking at follow-up (−0.482 [−0.925, −0.038]). Similarly, among baseline users (n = 647), PROH (binge drinking) partially mediated the cSBA effect (total effect −0.474 [−0.890, −0.058]; indirect effect −0.096, [−0.245, −0.016]), with cSBA enhancing PROH (0.204 [0.030, 0.378]) and higher PROH reducing odds of reporting drinking (−0.470 [−0.733, −0.207]).

**Conclusion:** A brief computer-facilitated primary care intervention can enhance adolescents’ perceived risk of harm from alcohol, which in turn contributes to reduction in short-term drinking rates.

## A3 Exploring patients’ experiences of alcohol screening and brief intervention delivery in English primary care using Normalization Process Theory

### Amy J. O’Donnell, Eileen Kaner, Barbara Hanratty

#### Institute of Health and Society, Newcastle University, Baddiley-Clark Building, Richardson Road, Newcastle upon Tyne, UK

##### **Correspondence:** Amy J. O’Donnell - amy.odonnell@newcastle.ac.uk

*Addiction Science & Clinical Practice* 2017, **12(Suppl 1)**: A3

**Background:** Various policy measures have been introduced to encourage alcohol screening and brief intervention (ASBI) implementation in English primary care, including clinical guidelines, financial incentives, and the incorporation of consumption questions in routine health checks. Whilst there is some evidence of the impact of such measures on GPs and other clinicians, we have little knowledge of the views of patients themselves. We used Normalization Process Theory (NPT) informed interviews to explore patients’ experiences of, and perspectives on, ASBI delivery in primary care.

**Materials and methods:** Semi-structured interviews with 22 patients who had discussed alcohol consumption in primary care. NPT-based topic guide focused discussions, with interviews audio-recorded and transcribed verbatim. Two-phase analysis: (1) framework analysis to identify emergent themes; (2) thematic mapping against core NPT constructs (coherence, cognitive participation, collective action, reflexive monitoring).

**Results:** We found mixed understanding of the adverse health consequences of excessive drinking amongst patients (coherence), with particularly limited appreciation of longer-term risks. There was some awareness of current alcohol guidelines but these were viewed flexibly, e.g. to accommodate increased consumption at special events. Screening usually took place within wider lifestyle discussions or new patient registrations; most described the experience as routine and not especially sensitive. Screen-positive patients were unaware of receiving an intervention for their drinking. Whilst patients enacted a range of strategies to limit alcohol consumption (collective action), often involving family or friends (reflexive monitoring), they viewed such strategies as learned through experience rather than based on expert clinician advice. However, despite skepticism around clinicians’ ability to elicit truthful information from patients, or stimulate positive change (especially with heavy drinkers, and against powerful socio-cultural influencers), there was support for primary care as a legitimate ASBI delivery setting (cognitive participation).

**Conclusions:** This study provides novel patient insights into alcohol prevention practice in England. We conclude there is strong acceptance of the screening role played by primary care clinicians but patients have less confidence in the effectiveness of alcohol interventions themselves. Alongside work to promote the benefits of structured alcohol lifestyle advice, there is a need to better communicate the advantages of drinking within lower risk limits.

## A4 Satisfaction, alliance and intervention experience: comparing provider- versus computer-delivered brief motivational interventions for substance use among childbearing aged women

### Amy M. Loree^1,2^, Kimberly A. Yonkers^3^, Steven J. Ondersma^4^, Kate Gilstead-Hayden^3^, Steve Martino^1,2^

#### ^1^VA Connecticut Healthcare System, West Haven, CT, USA; ^2^Department of Psychiatry, Yale University School of Medicine, New Haven, CT, USA; ^3^Department of Psychiatry, Yale University School of Medicine, New Haven, CT, USA; ^4^Merrill Palmer Skillman Institute and Department of Psychiatry and Behavioral Neurosciences, Wayne State University, Detroit, MI, USA

##### **Correspondence:** Amy M. Loree - amy.loree@yale.edu

*Addiction Science & Clinical Practice* 2017, **12(Suppl 1)**: A4

**Background:** Screening, brief intervention, and referral to treatment (SBIRT) delivered in person or electronically (eSBIRT) is recommended for identifying women who use substances and helping them reduce or discontinue their use. However, it is not known how eSBIRT compares to SBIRT with respect to patients’ experience of satisfaction and therapeutic alliance with brief interventions. It is also unknown if SBIRT and eSBIRT deliver similar components of a brief intervention. Our aim was to compare satisfaction and alliance ratings following receipt of SBIRT and eSBIRT and to compare intervention components received in both SBIRT groups.

**Materials and methods:** The present investigation used data collected as part of a multi-factorial randomized clinical trial (N = 439) comparing SBIRT, eSBIRT, and enhanced usual care for childbearing aged women receiving care in a reproductive health clinic. Participants were pregnant and non-pregnant women presenting for outpatient visits at an urban reproductive healthcare clinic who were at least 18 years of age. Participants in the SBIRT and eSBIRT groups completed satisfaction and alliance ratings following a single-session motivational intervention targeting substance use (items rated on a Likert scale, ranging from 1 strongly disagree to 7 strongly agree). Trained raters independently rated audio recorded SBIRT sessions for the presence of six major intervention components. Raters also rated the occurrence of these components in the eSBIRT program. Descriptive analyses and t-tests were used to examine differences between groups.

**Results:** Participants in both groups were very satisfied (M = 6.61 (0.57) for SBIRT; M = 6.36 (0.72) for eSBIRT) and felt allied (M = 6.79 (0.42) for SBIRT; M = 6.47 (0.62) for eSBIRT) with the intervention; though SBIRT participants were significantly higher in a few categories of each domain. Motivational intervention components received by each group were similar.

**Conclusions:** Findings suggest that participant satisfaction and alliance with SBIRT and eSBIRT are comparable, and that participants are exposed to similar intervention elements regardless of delivery method.

## A5 Acceptability and feasibility of the tobacco, alcohol, prescription medication, and other substance use (TAPS) tool in U.S. primary care patients

### Angeline Adam^1^, Robert P. Schwartz^2^, Li-Tzy Wu^3^, Geetha Subramaniam^4^, Gaurav Sharma^5^, Jennifer McNeely^1^

#### ^1^Department of Population Health, NYU School of Medicine, New York, NY, USA; ^2^Friends Research Institute, Inc., Baltimore, MD, USA; ^3^Department of Psychiatry and Behavioral Sciences, Department of Medicine and Duke Clinical Research Institute, Duke University Medical Center, Durham, NC, USA; ^4^Center for the Clinical Trials Network, National Institute on Drug Abuse, National Institutes of Health, Bethesda, MD, USA; ^5^The EMMES Corporation, Rockville, MD, USA

##### **Correspondence:** Angeline Adam - angeline.adam@nyumc.org

*Addiction Science & Clinical Practice* 2017, **12(Suppl 1)**: A5

**Background:** The TAPS Tool was developed as a brief substance use screening and assessment instrument for primary care. As part of a validation study of the TAPS Tool, we evaluated its acceptability to patients and feasibility of administration.

**Materials and methods:** Participants (N = 2000) recruited from five primary care clinics completed interviewer-administered (IA-TAPS) and computer self-administered (SA-TAPS) versions of the TAPS Tool. Time required and requests for assistance were recorded, and participants completed a 10-item questionnaire addressing user-friendliness, comfort, and format preference. We examined results for all participants and for subgroups: elderly (>65 years), lower education (<high school), alcohol/drug use, sex, race, and ethnicity.

**Results:** Almost all participants found the TAPS Tool easy to understand (99%), and said they would share results with their doctor (95%). 31% preferred the IA-TAPS, 38% the SA-TAPS, and 45% had no preference. The IA format was more frequently preferred by participants who were elderly (36 vs. 30%); less educated (49 vs. 26%); or used prescription drugs (34 vs. 30%). The SA format was preferred by participants who were African-American (40 vs. 35%); or used drugs (43 vs. 37%).

The mean time to complete IA-TAPS was 2.4 min; 90% of the participants completed in <3 min. The mean time to complete the SA-TAPS was 4.5 min: 90% of the participants completed in <7 min. The SA-TAPS was completed more slowly by participants who were elderly (mean 6.1 min) or lower education (mean 6.0 min). Assistance was requested by 8% for the IA-TAPS, and by 25% for the SA-TAPS. SA-TAPS assistance was most frequently requested by those who were elderly (48%), lower education (38%), or used prescription drugs (31%).

**Conclusions:** Both formats of the TAPS Tool were well accepted. The SA-TAPS was preferred by subpopulations who may experience more stigma related to substance use, while the IA-TAPS was preferred by those who may have more difficulty using a computer. The time required for the TAPS would be feasible in most primary care settings, but patients who are older or less educated may need assistance with the self-administered version.

## A6 Clinician experiences of healthy lifestyle promotion and perceptions of digital interventions as complementary tools for lifestyle behavior change in primary care

### Anne H. Berman^1^, Karoline Kolaas^1,2^, Elisabeth Petersén^1,3^, Preben Bendtsen^4^, Erik Hedman^1,2^, Catharina Linderoth^4^, Ulrika Müssener^4^, Kristina Sinadinovic^1^, Fredrik Spak^5^, Ida Gremyr^6^, Anna Thurang^1,3^

#### ^1^Department of Clinical Neuroscience, Center for Psychiatry Research, Karolinska Institutet, Stockholm, Sweden; ^2^Gustavsberg Primary Care Clinic, Odelbergs väg 19, Gustavsberg, Sweden; ^3^Stockholm Center for Dependency Disorders, Box 17914, Stockholm, Sweden; ^4^Department of Medicine and Health Sciences, Linköping University, Linköping, Sweden; ^5^Department of Social Medicine, Gothenburg University, Gothenburg, Sweden; ^6^Division of Service Management and Logistics, Chalmers Technological University, Sweden

##### **Correspondence:** Anne H. Berman - anne.h.berman@ki.se

*Addiction Science & Clinical Practice* 2017, **12(Suppl 1)**: A6

**Background:** Evidence-based healthy lifestyle promotion in primary health care has been supported internationally by national policies and guidelines but implementation in routine primary health care has been slow. Referral to digital interventions could lead to a larger proportion of patients accessing structured interventions for healthy lifestyle changes, but such referral might have unknown implications for clinicians with patients accessing such interventions. This qualitative study aimed to explore the perceptions of clinicians in primary care on healthy lifestyle promotion with or without digital screening and intervention.

**Materials and methods:** Focus group interviews were conducted at 10 primary care clinics in Sweden with clinicians from different health professions. Transcribed interviews were analyzed using a phenomenological-hermeneutic method involving naïve understanding, structural analysis and comprehensive understanding.

**Results:** Two major themes captured clinicians’ perceptions on healthy lifestyle promotion: (1) the need for structured professional practice and (2) deficient professional practice as a hindrance to implementation. Sub-themes in theme 1 were striving towards professionalism, which for participants meant working in a standardized fashion, with replicable routines regardless of clinic, as well as being able to monitor statistics on individual patient and group levels; and embracing the future with critical optimism, meaning expecting to develop professionally but also being concerned about the consequences of integrating digital tools into primary care, particularly regarding the importance of personal interaction between patient and provider. For theme 2, sub-themes were being in an unmanageable situation, meaning not being able to do what is perceived as best for the patient due to lack of time and resources; and following one’s perception, meaning working from a gut feeling, which for our participants also meant deviating from clinical routines.

**Conclusions:** In efforts to increase evidence-based practice and lighten the burden of clinicians in primary care, decision- and policy-makers planning the introduction of digital tools for healthy lifestyle promotion will need to explicitly define their role as complements to face-to-face encounters. Our overriding hope is that this study will contribute to maintaining meaningfulness in the patient-clinician encounter, when digital tools are added to facilitate patient behavior change of unhealthy lifestyle behaviors.

## A7 The integration of alcohol screening and brief intervention (aSBI) into nursing curricula: CDC/American Association of Colleges of Nursing - Workforce Improvement Projects (AACN-WIP) and other initiatives

### Ann M. Mitchell^1^, Deborah Finnell^2^, Christine L. Savage^3^, Khadejah F. Mahmoud^4^

#### ^1^Department of Health and Community Systems, University of Pittsburgh School of Nursing, Pittsburgh, PA, USA; ^2^Department of Acute and Chronic Care, Johns Hopkins University, Baltimore, MD, USA; ^3^School of Nursing, Johns Hopkins University, Baltimore, MD, USA; ^4^School of Nursing, University of Pittsburgh, Pittsburgh, PA, USA

##### **Correspondence:** Ann M. Mitchell - ammi@pitt.edu

*Addiction Science & Clinical Practice* 2017, **12(Suppl 1)**: A7

**Background:** Due to the need for nurses to have competency in the delivery of aSBI, it is important to address gaps in nursing curricula. The University of Pittsburgh School of Nursing has been infusing SBIRT education into its undergraduate and graduate curricula for years. Most recently, the CDC publication on planning and implementing alcohol screening and brief intervention provided the framework for the WIP program for nurse leaders including: clinical nurse leaders, nursing administrators, and nursing informaticists. The Johns Hopkins School of Nursing (01-117-WIPFA 14-JU) and the Pitt School of Nursing (02-117-WIPFA 14-UPITT) worked together to develop a 13-module, on-line aSBI curricula.

**Materials and methods:** The undergraduate curriculum includes 3-hour didactic, face-to-face SBIRT instruction, 15-weeks of clinical application, and infusion into the junior and senior years. The graduate curriculum includes didactic, face-to-face, and video instruction embedded throughout DNP courses including: history and physical exam, management of acute and chronic conditions, as well as adolescent, women, and older adult health courses; followed by clinical infusion through the program. Lastly, the curriculum for the nurse leaders builds on JHU and UPITT’s previous successes, enabling the two teams to collaborate on the development of a single product using Articulate Storyline, the premier e-learning development platform hosted on a learning management system.

**Results:** Education and clinical training had the most pronounced effect on indicators of Role Security including role adequacy (*p* < .05), role legitimacy (*p* < .05), and role support (*p* < .05) and Therapeutic Commitment including role specific-self esteem (*p* < .05), and work satisfaction (*p* < .05) for people who use alcohol in the undergraduate student nurses. It had significant positive results on role legitimacy (*p* < .05), motivation (*p* < .05), and work satisfaction (*p* < .05) for people who use alcohol in the graduate nurse practitioner students. Lastly, there was also a positive effect on education and attitudes for the nurse leader group.

**Conclusions:** These curricula infusion models provide easily accessible education and clinical training, bringing evidence-based aSBI/SBIRT to our current and future nurses. Education infused into multiple courses and levels of curricula can have positive effects on nurses’ Role Security and Therapeutic Commitment for providing care to individuals with alcohol use problems.

## A8 Intervening during the event-specific pregame: a text message intervention to reduce new students’ alcohol use during Orientation Week

### Benjamin C. Riordan, Tamlin S. Conner, Jayde A. M. Flett, Damian Scarf

#### Department of Psychology, University of Otago, Dunedin, New Zealand

##### **Correspondence:** Benjamin C. Riordan - ben.riordan@postgrad.otago.ac.nz

*Addiction Science & Clinical Practice* 2017, **12(Suppl 1)**: A8

**Background:** University students drink more during events than at any other time. One factor that may underlie the higher amount of alcohol consumed during events is pre-gaming (i.e., drinking before an event). In Study 1, we aimed to quantify the extent to which students’ pre-gamed before Orientation Week (O’Week) events using intercept surveys. In Study 2, we piloted a text message intervention targeting O’Week pre-gaming sessions, to determine whether we could reduce new students’ drinking during O’Week.

**Materials and methods:** In Study 1 we administered breathalysers and surveys to 335 students who were entering three O’Week events. Participants self-reported the number of drinks they had consumed and their Blood Alcohol Concentration (BAC) was recorded. In Study 2 we trialled a text message intervention with new students residing in two residential colleges (Dorm 1: n = 100, Dorm 2: n = 241) who were assigned to either a control or intervention condition. All students reported their O’Week drinking. Students in the intervention condition also received text messages before four O’Week events at 7:30 p.m. and 9 p.m. that pointed out the social harms of drinking using colloquial language.

**Results:** In Study 1, students consumed on average 5.7 drinks before the events with a mean BAC of 0.062. In Study 2, students in Dorm 1 receiving the intervention reported consuming significantly fewer drinks during O’Week than those in the control condition (intervention = 9.7 vs. control = 15.5; t(98) = 2.138, *p* = .018) despite reporting a similar amount of pre-university drinking (intervention = 5.8 vs. control = 6.4). However, there was no difference in drinking by Dorm 2 students at any time (O’Week: intervention = 36.7, control = 37.7, *p* = .768; pre-university: intervention = 13.4 vs. control = 16.0, *p* = .178).

**Conclusions:** Study 1 demonstrated that pre-gaming makes a substantial contribution to the amount of alcohol students consume during events. Study 2 revealed a text message intervention targeting pre-gaming reduced O’Week drinking in one dormitory but not another. One explanation for this finding is that the intervention was successful with the generally lighter drinkers in Dorm 1 but not the markedly heavier drinkers in Dorm 2.

## A9 The examination of three SBIRT implementation models

### Bonnie McRee, Janice Vendetti, Karen Steinberg Gallucci, Kate Robaina

#### Department of Community Medicine and Health Care, University of Connecticut School of Medicine, Farmington, CT, USA

##### **Correspondence:** Bonnie McRee - mcree@uchc.edu

*Addiction Science & Clinical Practice* 2017, **12(Suppl 1)**: A9

**Background:** The CT Screening Brief Intervention and Referral to Treatment (SBIRT) Program has employed three different models to implement services in 13 Federally Qualified Health Centers. Initially, the program utilized the Contracted Specialist or Health Educator (HE) model. HEs were supervised by an outside agency to remove the time constraints identified by the health care providers. Approximately two years later, the model shifted so that HEs became employees of and were supervised by the health centers. This model, the In-house Specialist, was intended to promote cohesion among the HE and health care team and to help sustain the program. To further address post-grant sustainability, a third model, the In-house Generalist, was utilized. In this model, in-house medical assistants were trained to administer the pre-screening tool and nurses or behavioral health staff conducted the full screen and provided the brief interventions, brief treatments or referrals to those screening positive for at-risk use.

**Materials and methods:** Screening data from the three models, 19 Contracted Specialists (HEs), 16 In-house Specialists, and 37 In-house Generalists over the course of the 5-year CT SBIRT program (2011–2016) were used to examine implementation model performance. Outcomes include the percentage of positive cases identified and substance use outcomes at 6-months following brief intervention.

**Results:** The Contracted Specialist Model (HEs) identified significantly more positive cases (16.7%) than did the In-house Specialist model (11.1%) or the In-house Generalist model (3.7%). For past 30-day substance use, in a subset of patients followed at 6 months, there were significant changes in “days of alcohol binge use,” “days of alcohol use,” or “days of marijuana use” compared to baseline days of use; however, there were no significant patient outcome differences across models.

**Conclusions:** The examination of the three models has implications for health policy and clinical practice. Dedicated HEs provide higher quality screening services by identifying at-risk patients at rates more consistent with those defined in the literature. Their intervention outcomes are similar to services provided by higher level, more costly staff. Provider reluctance to implement SBIRT services continues to be a major challenge. The need to explore alternative solutions is needed.

## A10 A critical illness recovery navigator for alcohol: a pilot study

### Brendan J. Clark^1^, Jacqueline Jones^2^, Kathryne D. Reed^1^, Rachel M. Hodapp^1^, Ivor Douglas^1^, Ellen L. Burnham^1^, Laura Aagaard^2^, Paul F. Cook^2^

#### ^1^Division of Pulmonary Sciences and Critical Care Medicine, School of Medicine, University of Colorado, Aurora, CO, USA; ^2^College of Nursing, University of Colorado, Aurora, CO, USA

##### **Correspondence:** Brendan J. Clark - brendan.clark@ucdenver.edu

*Addiction Science & Clinical Practice* 2017, **12(Suppl 1)**: A10

**Background:** Alcohol misuse is common in survivors of critical illness and is associated with an increased risk of morbidity and mortality. In this study, we adapted screening, brief intervention and referral to treatment (SBIRT) for survivors of critical illness to include motivational interviewing (MI) and shared decision making techniques. This adaptation was designed to address the challenges in building therapeutic alliance and autonomy and to address the need for referral to treatment in this population. We created a recovery navigator role to deliver this adapted intervention. We conducted a pilot study to assess acceptability and to ensure the feasibility and fidelity of the recovery navigator role.

**Materials and methods:** This non-randomized pilot study was conducted in two urban medical intensive care units. Men with an AUDIT-C score of 4 or greater or women with an AUDIT-C score of 3 or greater who were admitted to the medical ICU and who provided informed consent were enrolled. We assessed baseline acceptability using the Client Satisfaction Questionnaire-8 (CSQ-8), MI fidelity using the Motivational Interviewing Skill Code version 2.1 (MISC), and feasibility using the percentage of subjects who received at least one session with the recovery navigator and the percentage of patients who received at least one session after hospital discharge.

**Results:** We screened 37 and enrolled 8 patients over the course of 8 weeks. The median age of patients was 49 (range 31–66), 75% were male, and the median AUDIT score was 23 (range 12–33). Seven patients had an alcohol use disorder. The median CSQ-8 score was 32 (range 28–32), consistent with a high level of satisfaction. Each patient received at least one session with the recovery navigator (range 1–8) and six had at least one follow-up session after hospital discharge. Median MISC global ratings for acceptance, empathy, MI spirit and client self-exploration were 7 (range 5–7), 6 (range 4–7), 5 (range 4–6), and 6 (range 4–7), respectively; 98% of therapist utterances were MI consistent.

**Conclusions:** These preliminary results demonstrate that a critical illness recovery navigator can deliver an adapted, MI-based SBIRT intervention to patients starting in the hospital and continuing after hospital discharge.

## A11 Optimizing the impact of alcohol and drug screening and brief intervention among a high-risk population receiving services in New York City sexually transmitted disease clinics: a process and outcome evaluation of Project Renew

### Brett R. Harris^1^, Jiang Yu^2^, Margaret Wolff^3^, Meighan Rogers^4^

#### ^1^Department of Health Policy, Management, and Behavior, University at Albany School of Public Health, Rensselaer, NY, USA; ^2^Center on Addictions Research, University at Albany School of Social Welfare, Albany, NY, USA; ^3^Mount Sinai Icahn School of Medicine, New York, NY, USA; ^4^Bureau of STD Control, NYC Department of Health and Mental Hygiene, Long Island City, USA

##### **Correspondence:** Brett R. Harris - brh24@nycap.rr.com

*Addiction Science & Clinical Practice* 2017, **12(Suppl 1)**: A11

**Background:** Unhealthy substance use is associated with increased rates of sexually transmitted diseases (STDs), including HIV. In a high-risk STD clinic population in New York City (NYC), 30.5 and 16.5% reported a lifetime or current substance use disorder, respectively, yet only 1.4% were in treatment and 13.2% had ever been in treatment. Screening, brief intervention and referral to treatment (SBIRT) was first implemented in STD clinics in 2005, piloted in one NYC clinic, with the goal of reducing substance use and the associated risky behaviors which increase the risk STD acquisition. Building upon lessons learned, Project Renew represents the third iteration of SBIRT implementation in NYC STD clinics with the goal of expanding the reach of SBIRT services within and across STD clinics citywide and decreasing substance use, poor mental health, and risky behavior.

**Materials and methods:** SBIRT services were delivered February 2012-January 2015. Patients screening positive for substance misuse on the AUDIT and/or DAST-10 were provided a brief intervention and interviewed using the Substance Abuse and Mental health Services Administration Government Performance and Results Act data collection tool at baseline and six-month follow-up. Patients scoring in Zones 3–4 (AUDIT > 15 or DAST-10 > 2) were offered additional sessions of extended brief intervention (EBI). Service delivery was assessed using electronic medical records.

**Results:** 130,597 pre-screenings for risky substance use were conducted, 66,989 (51%) of which were positive leading to 17,474 brief interventions and 1138 referrals. Between baseline and follow-up, there was a 19.7 and 43.2% decrease in days of alcohol and drug use, respectively (*p* < .05). Greater decreases in use were seen among patients offered EBI (36.0 and 55.9% decrease in days of alcohol and drug use, respectively). Patients also self-reported reductions in number of sexual contacts and experienced fewer days of depression and anxiety (*p* < .05).

**Conclusions:** Project Renew successfully expanded the reach of services from previous project iterations and led to reductions in substance use, sexual risk behavior, and poor mental health which may help to prevent acquisition of HIV or other STDs. Based on positive results, services have been sustained under the ThriveNYC initiative, ensuring essential care to a large population of high-risk New Yorkers.

## A12 Cost-effectiveness of SBI implementation for adolescents

### Carolina Barbosa^1^, Brendan J. Wedehase^2^, Laura J. Dunlap^2^, Shannon G. Mitchell^3^, Kristi A. Dusek^3^, Robert P. Schwartz^3^, Jan Gryczynski^3^, Arethusa S. Kirk^4^, Marla T. Oros^5^, Colleen Hosler^5^, Kevin E. O’Grady^6^, Barry S. Brown^7^

#### ^1^Behavioral Health Economics Program, RTI International, Chicago, IL, USA; ^2^Behavioral Health Economics Program, RTI International, Research Triangle Park, NC, USA; ^3^Social Research Center, Friends Research Institute, Baltimore, MD, USA; ^4^Total Health Care, Baltimore, MD, USA; ^5^Mosaic Group, Towson, MD, USA; ^6^Department of Psychology, University of Maryland, College Park, MD, USA; ^7^Department of Psychology, University of North Carolina Wilmington, Wilmington, NC, USA

##### **Correspondence:** Carolina Barbosa - cbarbosa@rti.org

*Addiction Science & Clinical Practice* 2017, **12(Suppl 1)**: A12

**Background:** The cost-effectiveness of different implementation models of screening and brief intervention (SBI) delivery to adolescents has never been studied. A cluster randomized trial examined the implementation of adolescent SBI for substance use within a primary care setting in the US. Two different implementation models for conducting brief interventions (BIs) were compared: the Generalist Model (GM), with BIs provided by a primary care provider (PCP), and the Specialist Model (SM), with BIs provided by behavioral health personnel after hand-off by the PCP. Understanding how costs and outcomes vary by model is important for providers to plan for services, and for decision makers considering widespread implementation of SBI. We estimate the cost and cost-effectiveness of the two models of implementation.

**Materials and methods:** The cost of SBI services was calculated using an activity-based costing methodology. Cost collection instruments retrieved staff time spent in each delivery activity and quantity and type of non-labor resources. Effectiveness was measured as the percentage of patients receiving BI amongst those who needed it and retrieved from electronic medical records. Sensitivity analyses were conducted on the time and unit cost of hand-offs.

**Results:** The average cost of SBI per screen positive visit was $4 (SD $0.38) and $5 (SD $0.8) in generalist and specialist sites, respectively. The average time of a BI in the SM was more than triple that in the GM (18 vs. 5 min), and only 7% of patients needing a BI received one in the SM compared to 38% in the GM. In the SM, the hand-off process represented a significant use of resources that was not required in the GM. The GM economically dominated the SM as it was both more effective and less costly. In sensitivity analyses, there were situations where the GM was more effective but at an additional cost, making the GM more cost-effective for willingness-to-pay thresholds below $1 per one additional percentage increase in BI delivery.

**Conclusion:** The integration of behavioral health personnel into the BI delivery process in a primary-care setting providing general medical and behavioral health services to adolescents might not be a cost-effective approach.

## A13 Would universal delivery of Screening and Brief Intervention in primary care improve or exacerbate alcohol-related inequalities in health? A modelling study in England

### Colin Angus^1^, Sidney Sherborne^1^, Duncan Gillespie^1^, Amy O’Donnell^2^, Petra Meier^1^, Alan Brennan^1^

#### ^1^School of Health and Related Research, University of Sheffield, Sheffield, UK; ^2^Institute for Health and Society, University of Newcastle, Newcastle upon Tyne, UK

##### **Correspondence:** Colin Angus - c.r.angus@sheffield.ac.uk

*Addiction Science & Clinical Practice* 2017, **12(Suppl 1)**: A13

**Background:** National public health bodies in the UK have repeatedly advocated for a universal program of Screening and Brief Interventions (SBIs) in primary care. Alcohol is a major driver of socioeconomic inequalities in health and population-level interventions are commonly found to worsen inequalities unless specifically designed to avoid this. We aimed to quantify the likely impact of universal SBI delivery on health inequalities.

**Materials and methods:** We analyzed national survey data on alcohol consumption, primary care usage, AUDIT scores and self-reported SBI receipt, alongside hospital admissions and mortality records, to quantify the socioeconomic gradients in these outcomes. Results were combined using the Sheffield Alcohol Policy Model to estimate the long-term health impacts of universal screening and their distribution across the socioeconomic spectrum.

**Results:** Individuals in the most deprived social group drank 16.3% less on average, yet were 14.5% more likely to attend primary care and 84.0% more likely to screen positive on AUDIT than those in the least deprived group, after adjusting for sociodemographic factors. Rates of hospitalization and mortality due to alcohol were 4.2 and 3.0 times greater respectively in the most deprived groups. Combining these social gradients, we estimate that the most deprived group would receive 27.2% fewer interventions than the least deprived, but experience a 16.4% greater reduction in alcohol-related deaths.

**Conclusions:** Whilst a program of universal SBI delivery in primary care in England would lead to higher levels of intervention delivery in less deprived groups, the greatest health benefits would be seen in the most deprived groups. Universal screening would therefore narrow, rather than widen existing socioeconomic inequalities in health.

## A14 Effectiveness of a brief intervention group performed by nurses in addressing hazardous and harmful alcohol use

### Divane de Vargas, Janaina Soares

#### Department of Maternal-Infant and Psychiatric Nursing, University of São Paulo, School of Nursing, São Paulo, Brazil

##### **Correspondence:** Divane de Vargas - vargas@usp.br

*Addiction Science & Clinical Practice* 2017, **12(Suppl 1)**: A14

**Background:** In Brazil, 20% of those who consume alcohol fulfill criteria for hazardous/harmful use. This phenomenon contributes to a high prevalence of individuals who meet these criteria in the country’s health services, including in Primary Health Care (PHC) facilities. The objective of this study was to verify the effectiveness of a Brief Intervention Group (BIG) performed by nurses, in reducing the risky use of alcohol among PHC users.

**Materials and methods:** A randomized controlled trial was conducted at a PHC facility in Sao Paulo City, Brazil. The sample consisted of 180 individuals. All participants completed the Alcohol Use Disorders Identification Test (AUDIT); those with AUDIT scores between 8 and 19 were enrolled in the study; 44 participants completed all study phases. Individuals randomized to the experimental group were enrolled in the Brief Intervention Group (BIG), while individuals allocated to the control group received a flyer containing information about problems related to harmful alcohol consumption. Both groups participated in a follow-up review after 90 days. The BIG intervention consisted of four group sessions, with weekly meetings and a follow-up session after 90 days. A mixed linear model was used to evaluate the effectiveness of the BIG in reducing alcohol consumption.

**Results:** The experimental group had a statistically significant reduction (*p* ≤ 0.01) of about 10 points in the mean AUDIT score after BIG (before BIG = 15.89 ± 6.62-corresponding to risky use; after BIG = 6.40 ± 5.05-corresponding to low-risk use), while maintaining low-risk use (6.69 ± 6.38–low-risk use). The control group had a statistically significant reduction (*p* ≤ 0.01) of about 3 points in the mean AUDIT score after screening and feedback (baseline 13.11 ± 4.54-corresponding to risky use; 30-day follow-up score 9.83 ± 5.54-corresponding to risky use), and returned to the baseline pattern of alcohol use (13.00 ± 5.70-corresponding to risky use). The differences between the two groups were statistically significant (*p* ≤ 0.01).

**Conclusion:** The results suggest that brief intervention performed by nurses in-group in the context of the PHC was effective to reduce alcohol consumption among individuals with hazardous/harmful alcohol use.

## A15 Social determinants of health among emergency department patients who screen positive for unhealthy alcohol or drug use: implications for ED SBIRT

### Kelly M. Doran^1^, Donna Castelblanco^2^, Ian Wittman^2^, Donna Shelley^3^, John Rotrosen^4^, Lillian Gelberg^5^

#### ^1^Departments of Emergency Medicine and Population Health, NYU School of Medicine, New York, NY, USA; ^2^Department of Emergency Medicine, NYU School of Medicine, New York, NY, USA; ^3^Departments of Medicine and Population Health, NYU School of Medicine, New York, NY, USA; ^4^Department of Psychiatry, NYU School of Medicine, New York, NY, USA; ^5^Department of Family Medicine, David Geffen School of Medicine at UCLA, Department of Health Policy and Management, UCLA Fielding School of Public Health, Office of Healthcare Transformation and Innovation, VA Greater Los Angeles Healthcare System, Los Angeles, CA, USA

##### **Correspondence:** Kelly M. Doran - Kelly.Doran@nyumc.org

*Addiction Science & Clinical Practice* 2017, **12(Suppl 1)**: A15

**Background:** Emergency department (ED) patients have high levels of substance use as well as high levels of social needs that could impact health. Such social determinants of health (SDOH) may affect the success of ED SBIRT programs, yet little research has examined SDOH among substance using ED patients.

**Materials and methods:** We surveyed a random sample of ED patients at an urban, public hospital from November 2016–March 2017. Eligible patients were: ≥18 years old, medically/psychiatrically stable, not in police/prison custody, spoke English or Spanish, and had not already participated. RAs administered a 20–40 min survey. We used validated single-item screeners for current unhealthy alcohol and drug use (Smith et al. 2009, 2010). Questions on self-reported past 12 month social needs were taken from national surveys or prior studies.

**Results:** 782 patients participated. One-third (31.6%) screened positive for unhealthy alcohol use and 19.7% for any drug use. Rates of being unemployed or unable to work were 44.8% overall, 46.9% for those with unhealthy alcohol use (χ^2^ *p* = 0.43 for difference between those who did vs. did not screen positive), and 62.8% for those with drug use (*p* < .01). Homelessness rates (including living “doubled up”) in the past year were 21.2% overall, 30.0% for those with unhealthy alcohol use (*p* < .01), and 43.4% for those with drug use (*p* < .01). Inability to meet essential expenses was 42.7% overall, 43.0% for those with unhealthy alcohol use (*p* = 0.88), and 59.6% for those with drug use (*p* < .01). Telephone service was disconnected for 21.7% overall, 25.6% for those with unhealthy alcohol use (*p* = 0.11), and 29.8% for those with drug use (*p* = 0.02). Food insecurity rates were 52.9% overall, 57.5% for those with unhealthy alcohol use (*p* = 0.09), and 65.6% for those with drug use (*p* < .01).

**Conclusions:** ED patients have high rates of significant social needs, with higher rates found among patients with drug use. Unhealthy alcohol users had rates more similar to ED patients overall, with the exception of being more likely to experience homelessness. These findings suggest that ED SBIRT programs must recognize patients’ concurrent social needs, which might impact the effectiveness of interventions to address their substance use.

## A16 Integrated stepped care to address moderate alcohol use among HIV-positive patients with liver disease: results from a randomized clinical trial

### E. Jennifer Edelman^1,2^, Stephen A. Maisto^3^, Nathan B. Hansen^2,4^, Christopher J. Cutter^1^, Yanhong Deng^5^, James Dziura^5^, Lynn E. Fiellin^1,2^, Patrick G. O’Connor^1^, Roger Bedimo^6^, Cynthia Gibert^7^, Vincent C. Marconi^8^, David Rimland^8^, Maria C. Rodriguez-Barradas^9^, Michael S. Simberkoff^10^, Amy C. Justice^1,11^, Kendall J. Bryant^12^, David A. Fiellin^1,2^

#### ^1^Yale University School of Medicine, New Haven, CT, USA; ^2^Center for Interdisciplinary Research on AIDS, Yale University School of Public Health, New Haven, CT, USA; ^3^Syracuse University, Syracuse, NY, USA; ^4^College of Public Health, University of Georgia, Athens, GA, USA; ^5^Yale Center for Analytic Sciences, Yale University School of Public Health, New Haven, CT, USA; ^6^Veterans Affairs North Texas Health Care System and UT Southwestern, Dallas, TX, USA; ^7^D.C. Veterans Affairs Medical Center and George Washington University School of Medicine and Health Sciences, Washington, D.C., USA; ^8^Atlanta Veterans Affairs Medical Center and Emory University School of Medicine, Decatur, GA, USA; ^9^Michael E. DeBakey Veterans Affairs Medical Center and Baylor College of Medicine, Houston, Texas, Houston, TX, USA; ^10^VA NY Harbor Healthcare System and New York University School of Medicine, New York, NY, USA; ^11^VA Connecticut Healthcare System, Veterans Aging Cohort Study, West Haven, CT, USA; ^12^National Institute on Alcohol Abuse and Alcoholism HIV/AIDS Program, Bethesda, MD, USA

##### **Correspondence:** E. Jennifer Edelman - ejennifer.edelman@yale.edu

*Addiction Science & Clinical Practice* 2017, **12(Suppl 1)**: A16

**Background:** Among HIV-positive patients with liver disease, abstinence from alcohol consumption is recommended, yet is often not systematically addressed in HIV treatment settings. We sought to evaluate the effectiveness of integrated stepped care (ISC) versus treatment as usual (TAU) on alcohol abstinence.

**Materials and methods:** From January 2013 through July 2016, we conducted a 24-week randomized controlled trial at five Veterans Affairs Infectious Disease Clinics. Eligibility criteria included (1) moderate alcohol use—any alcohol consumption in the prior 30 days, but not meeting criteria for at-risk drinking or alcohol use disorder, and (2) liver disease—liver fibrosis (FIB-4 score >1.45) or detectable hepatitis C virus (HCV). Participants were randomized to ISC or TAU. ISC included: Step 1—Social Worker administered Brief Intervention with telephone booster; Step 2—Psychologist administered Motivational Enhancement Therapy over four sessions; and Step 3—Addiction Physician management with consideration of pharmacotherapy. Participants were “stepped up” to Step 2 and Step 3 if they reported any alcohol use in the prior 14 days at weeks 4 and 12, respectively. Generalized mixed effects models adjusted for baseline alcohol use and HIV disease severity, were used to examine past 28 day abstinence at week 24 assessed by Timeline Followback. Target enrollment was 228.

**Results:** Among 6391 AUDIT-C screening tests performed, 3265 exceeded zero. Of these, we enrolled 95 participants before stopping the trial due to under-enrollment. Ninety-nine percent were men, 85% black, 12% white, 4% Hispanic, with a mean age of 61 years. At baseline, 85% had a FIB-4 >1.45, 56% were HCV-infected. The median CD4 cell count was 853 cells/mm^3^ and 24% had a detectable HIV viral load. The mean (standard deviation) drinks per day was 0.49 (0.59). Compared to TAU, ISC was associated with non-significant increased odds of past 28 day abstinence at week 24 (adjusted odds ratio [95% confidence interval] = 3.20 [0.88, 11.67]).

**Conclusions:** Non-treatment seeking HIV-positive patients with moderate alcohol consumption and liver disease rarely enter trials to address their drinking. ISC holds promise as an intervention to promote abstinence in this population.

## A17 A multi-center individual-randomized controlled trial of screening and brief alcohol intervention to prevent risky drinking in young people aged 14–15 in a high school setting

### Emma L. Giles^1^, Dorothy Newbury-Birch^1^, Simon Coulton^2^, Paolo Deluca^3^, Colin Drummond^3^, Denise Howel^4^, Eileen Kaner^4^, Elaine McColl^4^, Ruth McGovern^4^, Stephanie Scott^4^, Elaine Stamp^4^, Harry Sumnall^5^, Luke Vale^6^, Viviana Alabani^6^, Amanda Atkinson^7^, Sadie Boniface^3^, Jennifer Ferguson^1^, Jo Frankham^8^, Eilish Gilvarry^9^, Nadine Hendrie^2^, Nicola Howe^10^, Grant J. McGeechan^1^, Amy Ramsey^3^, Grant Stanley^7^

#### ^1^School of Health and Social Care, Teesside University, Middlesbrough, Tees Valley, UK; ^2^Centre for Health Services Research, University of Kent, Canterbury, Kent, UK; ^3^Addictions Department, Institute of Psychiatry, Psychology and Neuroscience, King’s College London, London, UK; ^4^Institute of Health and Society, Newcastle University, Newcastle upon Tyne, UK; ^5^School of Education, Communication and Language Sciences, Newcastle University, Newcastle upon Tyne, UK; ^6^Health Economics Group, Institute of Health and Society, Newcastle University, Richardson Road, Newcastle upon Tyne, UK; ^7^Centre for Public Health, Liverpool John Moores University, Liverpool, UK; ^8^Faculty of Education, Health and Community, Liverpool John Moores University, Liverpool, UK; ^9^Northumberland, Tyne and Wear NHS Foundation Trust, Newcastle upon Tyne, UK; ^10^Newcastle Clinical Trials Unit, Newcastle University, Newcastle upon Tyne, UK

##### **Correspondence:** Emma Giles - e.giles@tees.ac.uk

*Addiction Science & Clinical Practice* 2017, **12(Suppl 1)**: A17

**Background:** Young people are a greater risk of problem drinking and are vulnerable to the effects of alcohol consumption, linked to early drinking and also the physiological and social consequences of alcohol use. The SIPS JR-HIGH study is a multicenter Randomized Controlled Trial (RCT) aiming to assess the effectiveness and cost-effectiveness of alcohol screening and brief intervention to reduce risky drinking in those aged 14–15 years in the English high school setting.

**Materials and methods:** Thirty schools in England were recruited into the trial: 4 in London; 6 in the North West, 7 in Kent, and 13 in the North East. A baseline survey was undertaken between December 2015 and June 2016 by young people in school. Young people who screened positive for risky drinking using a single item screen (ASAQ) were allocated with equal probability to either a control arm consisting of usual school-based education on alcohol issues, or to the intervention arm augmenting usual education with a 30 min brief intervention, both delivered by school pastoral staff. At 12-month follow-up the primary outcome of total alcohol consumed is being measured using the Timeline Follow Back (TLFB). Qualitative interviews with young people, parents, and school staff supplement the main trial, by exploring the barriers and facilitators to the effectiveness and implementation of the intervention.

**Results:** In total, 4587 young people were surveyed at baseline, with 586 randomized. So far, thirty-one (n = 109) percent of the target (n = 352) follow-up sample have completed a survey at their 12 month time point, with ongoing follow-ups continuing until June 2017. In total, 27 qualitative interviews with school staff were conducted in 2016, and interviews with young people and parents are currently being conducted.

**Conclusions:** Of 4587 young people surveyed, 1044 young people scored positive for risky drinking, and 586 are taking part in the trial. Qualitative interviews with school staff have indicated that they see the benefit of the intervention to schools, that there is staff support for the intervention, and that is easy to undertake by school staff and to roll-out in schools should it be shown to be effective and cost-effective.

## A18 Reducing risky drinking in children and young people: alcohol perceptions and attitudes interview and survey findings

### Emma L. Giles^1^, Justine Clephane^2^, Simon Coulton^3^, Jennifer Ferguson^1^, David Gardiner^4^, John Holmes^5^, Neil Martin^6^, Grant J. McGeechan^1^, Colin Shevills^6^, Melanie Soutar^7^, Dorothy Newbury-Birch^1^

#### ^1^School of Health and Social Care, Teesside University, Middlesbrough, Tees Valley, UK; ^2^Education Service, Wellbeing and Community Health Services Group, Brunel Building, 64 Regent Street, Blyth, UK; ^3^Centre for Health Services Research, University of Kent, Canterbury, Kent, UK; ^4^Public Health England North East, Waterfront 4, Goldcrest Way, Newburn Riverside, Newcastle upon Tyne, Tyne and Wear, UK; ^5^Section of Public Health ScHARR, University of Sheffield, Regent Court, 30 Regent Street, Sheffield, UK; ^6^Balance North East, Bede House, Durham, UK; ^7^Matrix Young People’s Service, 7 Burrow Street, South Shields, Tyne and Wear, UK

##### **Correspondence:** Emma Giles - e.giles@tees.ac.uk

*Addiction Science & Clinical Practice* 2017, **12(Suppl 1)**: A18

**Background:** Despite recent decreases in the number of children and young people (CYP) who drink alcohol, the North East (NE) of England has one of the highest youth drinking rates in the country with 10% of CYP drinking regularly. This research aims to identify factors which might impact on alcohol consumption: (1) intergenerational influences; (2) mimicking positive behaviors; (3) alcohol accessibility; and (4) social media and friendship.

**Materials and methods:** An online survey was undertaken by CYP at seven schools in the North East of England. The survey was emailed by schools to all of their CYP in Years 7–10 (aged 11–15 years) and was completed in early 2017, with a target sample size of 1200. It explored alcohol use in CYP, why they do and do not drink, and explored their wider environment which may impact on their alcohol intake. Following completion of the survey, semi-structured interviews targeting 30–50 CYP were undertaken to explore in more detail the issues raised in the survey.

**Results:** As of early March 2017, 760 CYP had completed the online survey, representing an average within school completion rate of 30%. In total, 32 in-depth interviews had also been undertaken with CYP from three schools. Final results for this study are expected at the end of April 2017. Preliminary results suggest that the majority of the sample do not currently drink alcohol. For those who do, they tend to consume alcohol on special occasions, such as birthdays, usually with parental approval. For those CYP that do not drink alcohol, they indicate that they perceive themselves to be too young to drink, that it is dangerous for health reasons, and that they wish to concentrate on their school performance. Also, they request more targeted information on alcohol.

**Conclusions:** The results have implications for policy and practice partners on this research, in ensuring current drug and alcohol campaigns are appropriately ‘pitched’ to CYP, and that CYP are receiving messages as intended. Reinforcing the factors that CYP identify as reasons for not drinking when targeting alcohol screening and brief interventions with CYP is a key consideration.

## A19 Racial/ethnic disparities in alcohol Screening, Brief Intervention and Referral to Treatment among adult hypertensive patients in primary care settings

### Felicia W. Chi^1^, Constance Weisner^1,2^, Thekla B. Ross^1^, Jennifer Mertens^3^, Stacy Sterling^1^

#### ^1^Division of Research, Kaiser Permanente Northern California, Oakland, CA, USA; ^2^Department of Psychiatry, University of California, San Francisco, San Francisco, CA, USA; ^3^Aurora Public Schools Division of Accountability and Research, Educational Services Center 1, Aurora, CO, USA

##### **Correspondence:** Felicia W. Chi - felicia.w.chi@kp.org

*Addiction Science & Clinical Practice* 2017, **12(Suppl 1)**: A19

**Background:** The high burden of unhealthy drinking and alcohol use disorders among patients with chronic conditions is well recognized. Alcohol Screening, Brief Intervention and Referral to Treatment (SBIRT) in adult primary care has been found efficacious in reducing hazardous drinking, and limited literature suggests positive effects of alcohol BI on blood pressure (BP) outcomes among hypertensive patients. This study examines whether there are racial/ethnic disparities in receipt of alcohol SBIRT among adult primary care hypertensive patients, by analyzing data of a clustered, randomized controlled trial on SBIRT implementation by primary care physicians (PCP arm) and non-physician providers (NPP and MA arm) in a large, integrated health care delivery system.

**Materials and Methods:** Electronic health record (EHR) data on 139,179 adult hypertensive patients who had a primary care visit at 36 clinics during the first year of the study were analyzed. We examined in each intervention arm, differences in demographic and clinical characteristics, screening rates, and BI/RT rates among those who screened positive, across racial/ethnic groups. Multilevel Logistic regressions further assessed the associations between race/ethnicity and receipt of alcohol SBIRT in each intervention arm while accounting for clustering of patients within physicians and clinics and adjusting for demographics, baseline severity, anti-hypertensive medication adherence and comorbidity.

**Results:** We found differences in demographic and clinical characteristics across racial/ethnic groups. Multilevel Logistic regressions found that compared to Whites, Asian/Pacific Islanders and Hispanics were more likely to receive screening in the PCP arm (adjusted Odds Ratios [95% confidence intervals] = 1.18 [1.07–1.30] and 1.12 [1.01–1.25], respectively), and African Americans and Hispanics were more likely to receive screening in the NPP and MA arm (adjusted Odds Ratios [95% confidence intervals] = 1.23 [1.01–1.50] and 1.19 [1.01–1.40], respectively). In both intervention arms, having uncontrolled BP was negatively associated with receiving screening for unhealthy drinking. However, no significant differences were found in receiving BI/RT when screened positive in either intervention arm. Interactions of race/ethnicity and gender were also examined.

**Conclusions:** Findings suggested comparable or higher SBIRT rates in non-White racial/ethnic groups compared to Whites among adult primary care hypertensive patients, although certain patient factors may play an important role for different SBIRT delivery models.

## A20 The variability of outcomes used in efficacy and effectiveness trials of alcohol brief interventions: a systematic review

### Gillian W. Shorter^1^, Nick Heather^2^, Emma L. Giles^1^, Aisha Holloway^3^, Jeremy Bray^4^, Anne H. Berman^5^, Amy J. O’Donnell^6^, Dorothy Newbury-Birch^1^

#### ^1^Alcohol and Public Health Team, School of Health and Social Care, Middlesbrough, Teesside, UK; ^2^Faculty of Health and Life Sciences, Northumbria University, Newcastle, Northumberland, UK; ^3^School of Health and Social Sciences, University of Edinburgh, Edinburgh, Midlothian, UK; ^4^Bryan School of Business and Economics, University of North Carolina at Greensboro, Greensboro, NC, USA; ^5^Department of Clinical Neuroscience, Center for Psychiatry Research, Karolinska Institutet, Stockholm, Sweden; ^6^Institute of Health and Society, Newcastle University, Newcastle upon Tyne, Northumberland, UK

##### **Correspondence:** Gillian W. Shorter - gillianwshorter@gmail.com

*Addiction Science & Clinical Practice* 2017, **12(Suppl 1)**: A20

**Background:** Evidence to evaluate the efficacy and effectiveness of alcohol brief interventions is weakened by variability in measured outcomes and inconsistent reporting. This ongoing systematic review forms part of the larger Outcome Reporting in Brief Intervention Trials: Alcohol (ORBITAL) project aligned with the INEBRIA special interest group of the same name. The review aims to identify outcomes and wider domains used in efficacy and effectiveness trials of alcohol brief interventions.

**Materials and Methods:** An ongoing systematic review and narrative synthesis of efficacy and effectiveness trials of alcohol brief interventions from 10 databases from Jan 2000–Sept 2016 (including EMBASE, MEDLINE, PsycINFO, CINAHL, Web of Science) and grey literature sources (including databases and trial registries). Alcohol brief intervention definitions are informed by National Institute of Clinical Excellence Public Health Guideline 24: Alcohol use disorders: prevention. The review was conducted in accordance with the Centre for Reviews and Dissemination (CRD) guidance and pre-registered on PROSPERO (CRD42016047185).

**Results:** From the initial search of databases around 320 studies were identified, with the grey literature search ongoing. In the first 95 studies included, there were seven different overarching domains used to summarize the outcomes; biomarkers, alcohol consumption (includes problems, hazardous, harmful, or risky drinking), economic factors and resource use, health measures, life impact, intervention factors, and psychological factors. Preliminary findings suggest the most commonly reported were consumption outcomes such as frequency of drinking (9.3% of 486 outcomes), frequency of heavy drinking (8.6%), typical drinks on occasion (10%) and number of drinks in a week (8.4%). Fewest reported included economic factors and resource use, life factors (such as quality of life), and biomarkers.

**Conclusions:** These preliminary findings indicate, as expected, considerable variability in the outcomes reported in alcohol brief intervention trials. Whilst it is perhaps unsurprising that consumption is the most common domain measured, there was considerable variability in how this is described as an outcome, and even more variability in how it is measured in practice. This illustrates the challenges in synthesizing the literature, and the need for a core outcome set to help alcohol brief interventionists to determine the minimum measurement standard for research and evaluation.

## A21 Research to practice: an evaluation of adolescent SBIRT training on student perceptions of confidence and attitudes toward implementing in the field

### Hildie A. Cohen^1^, Tracy L. McPherson^2^, Cyrille Adam^3^

#### ^1^NORC at the University of Chicago, Chicago, IL, USA; ^2^Public Health, NORC at the University of Chicago, Bethesda, MD, USA; ^3^Research and Development, Kognito, New York, NY, USA

##### **Correspondence:** Hildie A. Cohen - cohen-hildie@norc.org

*Addiction Science & Clinical Practice* 2017, **12(Suppl 1)**: A21

**Background:** Substance use in adolescence is linked to a range of negative life consequences. Consequently, there is a great need for social workers, nurses, and other health professionals to be trained in prevention and early intervention approaches to adolescent alcohol and marijuana use. To this end, NORC at the University of Chicago along with leading professional education associations and experts partnered to develop and test an adolescent screening, brief intervention, and referral to treatment (SBIRT) curriculum for use in nursing and social work education. The study aimed to evaluate the impact of the education implemented with more than 4000 students in 32 nursing and social work programs on students’ attitudes towards working with people who drink alcohol; perceived readiness, confidence, and competence; and skills.

**Materials and methods:** Students completed a pre-training evaluation survey, received adolescent SBIRT education including an online simulation training, and completed a post-training evaluation survey. A pretest–posttest within-subjects design was used to investigate the effects on student attitudes; confidence, competence, and readiness; and skills. Differences between groups were also explored for program-level variables. Through review of implementation progress reports and implementation teleconference calls, qualitative data was gathered to assess perceptions of how the education fit into curriculum at different program levels (e.g., bachelor, masters, doctoral).

**Results:** The adolescent SBIRT education was effective in improving a number of student outcomes assessed using the pretest–posttest evaluation survey of attitudes, confidence, competence, readiness, and skills. Differences by program level were also observed.

**Conclusions:** The implications of these findings suggest that adolescent SBIRT education including simulation-based training can positively affect student outcomes as they prepare to implement adolescent SBIRT in the field. The findings can also inform educators on the differences in outcomes among groups and inform curriculum infusion.

## A22 Don’t take it for granted

### Hugo López-Pelayo^1^, Antoni Gual^1^, Lidia Segura^2^, Joan Colom^2^

#### ^1^Grup de Recerca Addicions Clínic, Hospital Clínic de Barcelona, IDIBAPS. University of Barcelona, Barcelona, Catalonia, Spain; ^2^Program on Substance Abuse, Public Health Agency, Government of Catalonia, Barcelona, Catalonia, Spain

##### **Correspondence:** Hugo López-Pelayo - hlopez@clinic.cat

*Addiction Science & Clinical Practice* 2017, **12(Suppl 1)**: A22

**Background:** Facilitated access to digital brief intervention (e-BI) has been proposed as a strategy for overcoming well-known barriers of BI in primary care practice (e.g. lack of time or fear of patient reactions). However, the ODHIN study showed that facilitated access to e-BI has no impact on BI implementation. The lack of success of facilitated access to e-BI in this study might be explained by the time required for delivery of e-BI, (which may take as much time as brief oral advice) and by the insufficient training of general practitioners (GPs) in e-BI.

**Materials and methods:** A randomized controlled non-inferiority trial (e-BI versus face-to-face BI) of primary care-based facilitated access to an alcohol reduction website was conducted with 34 PCP’s of Catalonia (EFAR-Spain). A qualitative study on barriers and facilitators of e-BI was conducted with a subsample of the recruited GPs.

**Results:** One-hundred fifteen GPs were trained for this project, but only eight GPs (7%) achieved the original recruitment goal (10 patients per GP), and recruitment required twenty-four months instead of the twelve months initially planned. The qualitative study (on-line survey) is therefore underway, focusing on the participant GPs in EFAR-project Spain. We expect to identify specific barriers related to e-BI and the utility of e-BI for dealing with traditional barriers of BI (lack of training, time, specialized services to referred patients or incentives and risk of upsetting the patient).

**Conclusion:** Our hypothesis is that facilitated access to e-BI does not sort out all the barriers to providing BI in primary care practice and adds new barriers that we should take into account in order to properly implement facilitated access. The results of the qualitative study should help to reframe our facilitated access strategies.

## A23 Vida PURA: results from a pilot randomized trial of screening and brief intervention with Latino day laborers

### India J. Ornelas^1^, Suzanne Doyle^2^, Dennis Donovan^2^, Bonnie Duran^3^, Vanessa Torres^1^

#### ^1^Department of Health Services, University of Washington, Seattle, WA, USA; ^2^Alcohol and Drug Abuse Institute, University of Washington, Seattle, WA, USA; ^3^School of Social Work, University of Washington, Seattle, WA, USA

##### **Correspondence:** India J. Ornelas - iornelas@uw.edu

*Addiction Science & Clinical Practice* 2017, **12(Suppl 1)**: A23

**Background:** Vida PURA is a culturally adapted intervention that consists of promotores providing screening and brief intervention at a day labor worker center to reduce unhealthy alcohol use among Latino day laborers.

**Materials and methods:** We conducted a pilot randomized control trial to test the efficacy of the Vida PURA intervention. Participants were screened for eligibility using the AUDIT (n = 181). Those with an AUDIT score ≥6 completed a baseline survey (n = 121) and were randomized into an intervention (n = 71) or control group (n = 30). Participants in the intervention group received a brief intervention from a promotor at a day labor worker center. Personalized feedback was provided by promotores, using a tablet screen to display the participants’ quantity of daily and weekly drinking. We conducted follow-up surveys at eight weeks following the baseline to assess changes in AUDIT scores, daily and weekly drinking.

**Results:** At baseline, mean AUDIT scores were 19.1 for men in the intervention group (n = 71) and 21.5 for those in the control group (n = 30). Both groups had decreased their AUDIT scores at eight weeks (intervention, 15.6; control, 18.2) with no significant differences between groups. Both groups also decreased their average number of drinks per drinking day from baseline to eight weeks (intervention, 2.9–1.8; control, 4.5–3.8, *p* < .05). Number of drinking days in the past two weeks also decreased in both groups from baseline to eight weeks (intervention, 5.7–4.2; control, 7.1–6.5, *p* < .05).

**Conclusions:** Given that there were no significant differences across groups, discussing alcohol use with a promotor during the survey may have been enough to initiate changes in drinking behaviors among participants. Future research should assess appropriate interventions for reducing unhealthy alcohol use in this population.

## A24 Developing a brief motivational intervention for young adults intoxicated in the ED: results from an iterative qualitative design

### Jacques Gaume, Véronique Grazioli, Cristiana Fortini, Sophie Paroz, Nicolas Bertholet, Jean-Bernard Daeppen

#### ^1^Alcohol Treatment Center, Lausanne University Hospital, Lausanne, Switzerland

##### **Correspondence:** Jacques Gaume - jacques.gaume@chuv.ch

*Addiction Science & Clinical Practice* 2017, **12(Suppl 1)**: A24

**Background:** Harmful alcohol use among young adults is a major public health concern. In Switzerland, Emergency Department (ED) admissions for alcohol intoxication have increased substantially over the past decade, particularly among adolescents and young adults. Brief motivational interventions for young adults in the ED have shown promising but inconsistent results.

**Materials and methods:** Based on the literature on brief intervention and motivational interviewing efficacy and active ingredients, we developed a new motivational intervention model for young adults admitted in the ED with alcohol intoxication. Using an iterative qualitative design, we first pre-tested this model by conducting 4 experimental sessions to evaluate interventionists’ and patients’ experience, then conducted a consultation with 9 international experts using nominal group technique, then re-tested the model by conducting 6 experimental sessions to evaluate interventionists’ and patients’ experience. At each round, data collected were analyzed and discussed, and the intervention model updated accordingly.

**Results:** Based on the literature, we found 6 axes for developing a new model: High level of relational factors (e.g. empathy, alliance, avoidance of confrontation); Personalized feedback; Enhance discrepancy; Evoke change talk while softening sustain talk, strengthen ability and commitment to change; Completion of a change plan; Devote more time: longer sessions and follow-up options (face-to-face, telephone, or electronic boosters; referral to treatment). Qualitative analysis of experimental sessions gave important insights regarding acceptability and feasibility of the model. Refinement comprised which feedback and information to provide and how, as well as how to deal with change planning with patients having vague change objectives. Experts’ consultation addressed numerous points, including reflections on information and advice giving, as well as follow-up interventions.

**Conclusions:** This iterative, multi-component design allowed developing an intervention model embedded in recent research findings and theory advances, as well as feasible in a complex environment. Next step is a randomized controlled trial testing the efficacy of this model.

## A25 Computer-facilitated 5A’s for tobacco use disorders: using technology to improve screening and brief interventions

### Jason M. Satterfield^1^, Steven Gregorich^1^, Nicholas J. Alvarado^1^, Ricardo Muñoz^2^, Gozel Kulieva^1^, Maya Vijayaraghavan^3^

#### ^1^Department of General Internal Medicine, University of California San Francisco, San Francisco, CA, USA; ^2^PhD Program in Clinical Psychology, Palo Alto University, Palo Alto, CA, USA; ^3^Department of Medicine, University of California San Francisco, San Francisco, CA, USA

##### **Correspondence:** Jason M. Satterfield - Jason.Satterfield@ucsf.edu

*Addiction Science & Clinical Practice* 2017, **12(Suppl 1)**: A25

**Background:** Clinical practice guidelines recommend that primary care providers (PCPs) deliver the 5A’s (ask, advise, assess, assist, and arrange) at every clinical encounter for the treatment of tobacco use disorders. Unfortunately, while most clinicians “ask” and “advise,” adherence to the “assist” and “arrange” steps remains low due to time and skill limitations. Innovative service delivery models are needed to improve 5A’s adherence.

**Objective:** To evaluate effectiveness of a computer-facilitated 5A’s (CF-5A’s) intervention to improve PCP 5A’s adherence. Primary outcomes include adherence to each “A” and to the 5A’s as a whole.

**Materials and methods:** PCPs from 3 clinics (HIV, safety net, and academic) were randomized into the CF-5A’s intervention or to usual care (UC). Adult patients who smoke were recruited in waiting rooms and assigned to their provider’s condition. Intervention patients completed the CF-5A’s and two tailored clinical summaries were generated—one for the provider and one for the patient. UC patients completed an eligibility survey and consent only. Within 72 h of the appointment, patients completed a post-visit survey about their receipt of the 5A’s during their PCP encounter. Patients could participate up to three times within the yearlong study period.

**Results:** N = 221 providers saw n = 961 patients (n = 412 intervention; n = 549 UC) in n = 1340 total encounters with n = 1011 completed post surveys (75.4% response). After accounting for 4-level nesting effects, GEE models showed intervention PCPs 32% more likely to “Assess” (OR 1.32; 95% CI, 1.01–1.72), 45% more likely to “Assist” (OR 1.45; 95% CI, 1.08–1.93), and 72% more likely to “Arrange” in the first visit only (OR 1.72; 95% CI, 1.23–2.40), and 104% more likely to complete all 5A’s during the first visit (OR 2.04; 95% CI, 1.35–3.07).

**Conclusion:** The CF-5A’s model improved PCP’s 5A’s adherence. Effectiveness was attenuated by clinic site and affected by the number of visits with earlier visits showing stronger results. While this low cost intervention has great potential for improving the implementation and delivery of tobacco cessation and other services, future studies should identify ways to promote and sustain technology implementation and integration with clinic procedures.

## A26 A tablet-based device for substance use and physical activity screening: spontaneous use in primary care waiting rooms

### Jean-Bernard Daeppen^1^, Angéline Adam^2^, John A. Cunningham^3^, Nicolas Bertholet^1^

#### ^1^Department of Community Medicine, Lausanne University Lausanne, Switzerland; ^2^Alcohol Treatment Center, Lausanne University Hospital, Lausanne Switzerland; ^3^Centre for Addiction and Mental Health, Toronto, Canada

##### **Correspondence:** Jean-Bernard Daeppen - jean-bernard.daeppen@chuv.ch

*Addiction Science & Clinical Practice* 2017, **12(Suppl 1)**: A26

**Background:** Screening and brief intervention for unhealthy substance use in primary care is challenging. Electronic devices may help clinicians to deliver screening and brief interventions to their patients, but spontaneous use in waiting rooms may be limited.

**Materials and methods:** We developed a tablet-based device specifically designed for primary care practices waiting rooms. The device offers screening for tobacco, illicit drugs, prescription drugs, and physical activity. Those screening positive for unhealthy alcohol use have the option of completing an electronic brief intervention. In February 2017, we recorded the number of patients attending 4 primary care practices, the number of patients completing the screening, and screening results. On random half-days, a research assistant was present to offer patients to use the device, allowing for comparison between spontaneous and assisted use of the device. The other days, a poster in the waiting room invited the patients to use the device.

**Results:** Out of 1781 patients attending the 4 practices, 342 (19.2%) used the device. Spontaneous use was lower (243 completed screen out of 1501 patients, 16.2%), compared to use assisted by a research assistant (99 completed screen out of 280 patients, 35.4%). Data indicated a profile of heavier severity for patients with spontaneous use, compared to counterparts, being younger (44.5 [17.1 vs. 49.9 [16.9], *p* = .009), more likely to smoke cigarettes (41.3 vs. 29.6%, *p* = .04), and use drugs (11.5 vs. 4.1%, *p* = .04), respectively. No statistically significant group differences were observed regarding proportion of patients with unhealthy alcohol use (58.4 vs. 49.0%), prescription drug use (26.3 vs. 16.7%) and reporting insufficient physical activity (52.3 vs. 47.9%). Spontaneous use was associated with a 54.5% completion of an electronic alcohol brief intervention (45.8% completion with assisted use).

**Conclusions:** Spontaneous use was lower compared to assisted use and appeared to self-select patients with heavier tobacco and drug use who appear more likely to use the device.

## A27 Alcohol screening and brief interventions with male remand prisoners: a cross sectional survey of prevalence, feasibility and acceptability

### Jennifer Ferguson^1^, Dorothy Newbury-Birch^1^, Aisha S. Holloway^2^

#### ^1^Health and Social Care Institute, School of Health and Social Care, Teesside University, Middlesbrough, Tees Valley, UK; ^2^Nursing Studies, School of Health in Social Science, The University of Edinburgh, Edinburgh, UK

##### **Correspondence:** Jennifer Ferguson - jennifer.ferguson@tees.ac.uk

*Addiction Science & Clinical Practice* 2017, **12(Suppl 1)**: A27

**Background:** In the UK, a significant proportion of male remand prisoners have alcohol problems. Alcohol Brief Interventions (ABIs) are an effective component of a population-level approach to harmful and hazardous drinking. ABI’s have been shown to reduce the aggregate level of alcohol consumed and therefore to reduce harm to the individual and to others. However, in relation to remand prisoners, there is no evidence as to how effective ABI’s could be. The aims of this study were therefore to explore the feasibility and acceptability of an ABI for adult male remand prisoners, and to develop an ABI for this group to be piloted in a future trial. This presentation presents the findings from the cross-sectional survey part of the study.

**Materials and methods:** A cross-sectional survey of adult male remand and convicted prisoners (n = 502) was carried out at one Scottish prison and one English prison to assess prevalence of alcohol use disorders. The questionnaire also included questions related to prisoners’ views on the acceptability and feasibility of ABIs in the prison system.

**Results:** 502 surveys across the two sites were completed by remand and convicted prisoners. Of these, 79% scored positive on the AUDIT (8+), with 44% of prisoners scoring as probably dependent (20+). Of all prisoners, 37% thought 5 min of advice would be useful, and 51% thought 20 min of advice would be useful. Eighty-six percent said they would be willing to take part in a future ABI effectiveness study to test and to be followed-up to collect post-test data. Forty-seven percent said they did not feel pressurized to take part in a research study whilst detained in prison.

**Conclusions:** Prevalence rates of risky drinking are higher in the criminal justice system compared to the general population. Prisoners were generally accepting of screening and brief interventions in this setting.

## A28 Qualitative evaluation of the drink-less project

### Joan Colom^1^, Lidia Segura-Garcia^1^, Estela Díaz^1^, Jorge Palacio-Vieira^1^, Antoni Gual^2^

#### ^1^Program on Substance Abuse, Public Health Agency of Catalonia, Barcelona, Spain; ^2^Addiction Unit, Hospital Clínic, Barcelona, Spain

##### **Correspondence:** Joan Colom - joan.colom@gencat.cat

*Addiction Science & Clinical Practice* 2017, **12(Suppl 1)**: A28

**Background:** After more than 20 years of research, evidence still shows that BI is effective in reducing alcohol quantity consumed in primary health care (PHC) (Platt et al., 2016). In Catalonia, in the framework of PHASE IV of the WHO Collaborative Project on Detection and Management of Alcohol-related problems in PHC (Heather et al., 2004), we developed a country-wide strategy and have been implementing it under the principles of action research over the last 15 years. Here we will describe the core implementation strategy components at all levels, and present the results of the evaluation undertaken and the resulting decisions made to achieve an enduring and routine implementation.

**Materials and methods:** A cross-sectional observational study simultaneously combining quantitative (systematic data of EIBI implementation rates from the PHC monitoring system) and qualitative methodologies (semi-structure interviews, survey, etc.) was undertaken. A convenience sample was used.

**Results:** A total of 33 professionals were invited to participate (60% family doctors, 25% nurses and 15% health technicians). The majority were alcohol referents and 6 rejected. 84% of the professionals believe that the program has clearly contributed to the increase in the detection of risky drinkers, as confirmed by the clear increase of more than 35% reported by the monitoring system. 20% believe that it has contributed to facilitating and improving the relationship between PHC and Addiction specialist centers. Among the main weaknesses found: low professional motivation, lack of time, alcohol not a priority, low incentives, high rotation, prejudices from professionals in front of patients with alcohol problems, and poor monitoring and feedback to professionals. Among the main strengths found: quality of training, comprehensive program, program sustainability, computerized screening tools in the medical record, strong alliances with societies, strong alcohol referent network, high visibility of the program, and availability of referral to treatment.

**Conclusions:** Changes in PHC are rather slow and require continuous sustainability actions. Organizational changes such as more time for preventive activities, CME, and empowerment through incentives and accreditation of the alcohol referent figure, and more patient-targeted raising awareness campaigns are key in order to overcome barriers.

## A29 Can Amazon’s mechanical turk be used to recruit participants for Internet intervention trials? A pilot study involving a randomized controlled trial of a brief online intervention for hazardous alcohol use

### John A. Cunningham, Alexandra Godinho, Vladyslav Kushir

#### Centre for Addiction and Mental Health, Toronto, Ontario, Canada

##### **Correspondence:** John A. Cunningham - john.cunningham@camh.ca

*Addiction Science & Clinical Practice* 2017, **12(Suppl 1)**: A29

**Background:** Amazon’s Mechanical Turk is a crowdsourcing platform that has been used extensively to collect psychological survey data. This pilot study sought to evaluate whether MTurk might also be a viable means of recruiting participants for online intervention research.

**Materials and methods:** Participants were recruited to complete an online survey about their alcohol use through the MTurk platform. Those who met eligibility criterion for problem drinking were invited to complete a 3-month follow-up. Of those who agreed, a randomized half were asked to access an online brief intervention for drinking (CheckYourDrinking.net) and the other half were assigned to a no intervention control group (i.e., thanked and told that they would be re-contacted in 3 months).

**Results:** A total of 423 participants were recruited, of which 85% were followed-up at 3-months. Only 1/3 of participants asked to access the online brief intervention did so. There was no significant difference between groups on the primary outcome variable—number of drinks in a typical week. One of three secondary outcome variables (AUDIT-C, highest number on one occasion, number of consequences experienced) displayed a significant difference between condition, with those being asked to access the online intervention reporting significantly greater reductions in AUDIT-C scores at follow-up compared to participants in the control condition (*p* = .004).

**Conclusions:** Despite the current pilot showing only limited evidence of impact of the intervention among participants recruited through MTurk, there is potential for conducting trials employing this population (particularly if methods are employed to make sure that participants receive the intervention). This potential is important as it could allow for the rapid conduct of multiple trials during the development stages of online interventions.

## A30 A brief intervention for alcohol use with suicidal adolescents in inpatient psychiatric treatment

### Kimberly H. M. O’Brien^1^, Laika D. Aguinaldo^2^, Christina M. Sellers^3^, Anthony Spirito^4^

#### ^1^Department of Health and Human Development, Education Development Center, Waltham, MA, USA; ^2^Department of Social Work, The Ethelyn R. Strong School of Social Work at Norfolk State University, Norfolk, VA, USA; ^3^Department of Social Work, Boston College School of Social Work, Chestnut Hill, MA, USA; ^4^Department of Psychiatry and Human Behavior, Brown University, Providence, RI, USA

##### **Correspondence:** Kimberly H. M. O’Brien - kobrien@edc.org

*Addiction Science & Clinical Practice* 2017, **12(Suppl 1)**: A30

**Background:** Substance use assessment and counseling is frequently neglected in inpatient psychiatric units when the adolescent’s suicide risk is the primary focus of treatment. Given the potential role alcohol could play in subsequent suicidal behaviors, greater attention to alcohol use in inpatient psychiatric treatment is critical. The purpose of this study was to test the feasibility and acceptability of a brief alcohol intervention with suicidal adolescent inpatients reporting past month drinking, and assess preliminary effects on alcohol use.

**Materials and methods:** In the trial, 39 adolescents (M_age_ = 15.63; 80% female, 65% white) and their families were recruited from an urban inpatient psychiatric hospital and randomly assigned to the experimental intervention (EXP) or treatment as usual (TAU), stratifying by gender and frequency of alcohol use. At baseline and 3 month follow-up, alcohol use was measured by the Timeline Follow-back Interview. Adolescents randomized to EXP received an individual session to explore alcohol use as a risk factor for continued suicidal behaviors and create a change plan, and a subsequent family session to discuss the change plan and strengthen the adolescent’s commitment and self-efficacy as well as the parent’s ability to support the adolescent. Adolescents in EXP were given an exit interview and session evaluation form to assess the intervention feasibility and acceptability.

**Results:** Of the adolescents, 19 were randomized to EXP and 20 to TAU. All 19 in EXP completed the individual intervention (M = 75 min) and family intervention (M = 20 min) during their inpatient hospitalization. All 19 expressed satisfaction with the intervention, and all 19 created a change plan. Adolescents in EXP drank less alcohol at 3 month follow-up (M = 11.69, SD = 28.47) relative to baseline (M = 41.56, SD = 43.40), Z = −2.90, *p* < .05. Adolescents in TAU also drank less at follow-up (M = 6.37, SD = 14.58) compared to baseline (M = 30.90, SD = 53.75), Z = −3.46, *p* < .05.

**Conclusions:** Results indicated that a brief alcohol intervention is feasible and acceptable to psychiatrically hospitalized suicidal adolescents, and may help to reduce their amount of alcohol use at 3 month follow-up. A larger fully powered study with a longer follow-up period is needed to test intervention effects and potential moderators.

## A31 An evaluation of screening measures to detect substance use in pregnancy

### Kimberly A. Yonkers^1^, Steven J. Ondersma^2^, Grace Chang^3^, Tiffany Blake-Lamb^4^

#### ^1^Department of Psychiatry, Yale School of Medicine, New Haven, CT, USA; ^2^Department of Psychiatry and Behavioral Neurosciences and Merrill-Palmer Skillman Institute, Detroit, MI, USA; ^3^Department of Psychiatry, VA Boston Healthcare and Harvard University, Boston, MA, USA; ^4^Department of Obstetrics and Gynecology, Massachusetts General Hospital, Boston, MA

##### **Correspondence:** Kimberly A. Yonkers - Kimberly.Yonkers@Yale.edu

*Addiction Science & Clinical Practice* 2017, **12(Suppl 1)**: A31

**Background:** In 2016, there were over 3.9 million births in the US. The 2013 National Survey on Drug Use and Health suggests that from this group about 5% used illicit substances, 9% consumed alcohol and 15% smoked nicotine cigarettes. A five-fold increase in antepartum maternal opiate use, coincident with an “epidemic” of opiate prescription abuse between 2000 and 2009 is also reported. These substances are harmful to mother and potentially her offspring, rendering the identification of women who use hazardous substances in pregnancy a public health priority. Unfortunately, only a few screening tools have been evaluated in pregnant women and none were tested against biochemical assays of substance use such as a urine toxicology test. The goal of this project was to compare the performance of five screening tools for detection of substance use in women to a urine toxicology screen.

**Materials and Methods:** Four existing screening tools with published evidence regarding detection of drug use in pregnancy (SURP-P, CRAFFT, WIDUS, and 5Ps), plus the NIDA Quick Screen, were administered to pregnant women age 18 or over who were recruited from one of three sites. Screening tools were administered in counterbalanced order and were followed by collection of a urine sample that was tested for illicit and licit substances.

**Results:** The cohort included 1198 pregnant women, 38% of whom were African-American and 15.5% of whom were Latina; 35% of participants had a Bachelor’s degree or higher, and 188 (15.9%) had a urine test positive for drugs or alcohol. Assessments were evenly distributed across trimesters. The overall accuracy in identification of a positive urine screen was 83.8% for the NIDA Quick Screen, 73.9% for the WIDUS, 70.5% for the SURP-P, 68.7% for the CRAFFT, and 42.5% for the 5Ps. Sensitivity, specificity, and positive and negative predictive values for the NIDA Quick Screen were 49.1, 90.0, 47.0, and 90.8, respectively.

**Conclusions:** The parsimonious NIDA Quick Screen showed good accuracy but poor sensitivity in predicting urine drug screen results. Future analyses will consider potential new screening tools made from existing items, multi-step screening, and gold standards taking calendar-based recall and/or diagnostic status into account.

## A32 Implementing SBIRT for youth and young adults in primary care: the New Hampshire Youth SBIRT initiative

### Lea R. Ayers LaFave^1^, Kathleen M. Thies^2^, Amy L. Pepin^1^, Kara E. Sprangers^1^, Martha Bradley^1^, Shasta Jorgensen^1^, Nico A. Catano^1^, Adelaide R. Murray^1^, Deborah Schachter^3^

#### ^1^JSI Research and Training Institute, Inc., Bow, NH, USA; ^2^TLQ Associates Healthcare Consulting LLC, Bedford, NH, USA; ^3^New Hampshire Charitable Foundation, Concord, NH, USA

##### **Correspondence:** Lea R. Ayers LaFave - llafave@jsi.com

*Addiction Science & Clinical Practice* 2017, **12(Suppl 1)**: A32

**Background:** Screen-Brief Intervention-Referral to Treatment (SBIRT) in pediatric practices normalizes conversations between youth and health care providers about alcohol and substance use, and supports guidance about healthy behaviors. SBIRT also identifies youth ages 12–22 whose current use of addictive substances places them at risk for developing substance use disorders, prompting providers’ brief intervention and referral for further assessment or treatment before substance use disorders develop.

**Materials and methods:** From May 2014 to June 2017, SBIRT was implemented as a standard of care in 23 pediatric practices in three cohorts across 10 organizations in New Hampshire—including academic medical centers and FQHCs—serving over 25,000 youth. Sites adapted either the CRAFFT or S2BI screening tools for their electronic health record (EHR). Materials and methods included developing a playbook for implementing SBIRT, training in Brief Intervention (BI) for 174 providers, and technical assistance provided on site, by phone, and email, and during scheduled meetings and video conferences, to support changes in office workflow, and integration of screening tools into EHRs for billing, documentation and data collection.

**Results:** While data collection will be completed in June 2017, the goal of 10,000 youth screened was exceeded in December 2016, with most sites sustaining screening rates above 85%. About 18% of youth were identified as at risk. Although 29% of them needed referral to behavioral health treatment, fewer than one-third received referrals from their providers, either because they were already in treatment, or because parents or the patient refused. Practices reported developing relationships with other service providers as an outcome of this work. Challenges included difficulties collecting data from EHRs, and inconsistent insurance reimbursement policies for screening and BI, and follow-up with youth referred for behavioral health treatment.

**Conclusions:** Pediatric practices can readily incorporate SBIRT into routine screening protocols. Providers reported that SBIRT helped them to normalize conversations about drugs and alcohol as part of routine pediatric practice, and that patients were surprisingly open to further conversation. Closing the loop with youth referred for treatment remains a challenge.

## A33 A pilot replication of QUIT, a randomized controlled trial of a brief intervention for reducing risky drug use, among Latino primary care patients

### Lillian Gelberg^1^, Ronald M. Andersen^2^, Guillermina Natera Rey^3^, Mani Vahidi^1^, Melvin W. Rico^1^, Sebastian E. Baumeister^4^

#### ^1^Department of Family Medicine, David Geffen School of Medicine at UCLA, Los Angeles, CA, USA; ^2^Fielding School of Public Health, University of California, Los Angeles, Los Angeles, CA, USA; ^3^National Institute of Psychiatry Ramón de la Fuente Muñiz, Mexico City, Mexico; ^4^Department of Epidemiology and Preventive Medicine, University of Regensburg, Regensburg, Germany

##### **Correspondence:** Lillian Gelberg - lgelberg@mednet.ucla.edu

*Addiction Science & Clinical Practice* 2017, **12(Suppl 1)**: A33

**Background:** QUIT is the only primary care-based brief intervention that has previously shown efficacy for reducing risky drug use in the US (Gelberg et al., 2015). This pilot study replicated the QUIT randomized controlled trial in one of the five original QUIT clinics that primarily serves Latinos.

**Materials and Methods:**

Design: Single-blind two-arm randomized controlled trial of patients enrolled from March–October 2013 with 3-month follow-up. Setting: Primary care waiting room of a FQHC in East Los Angeles. Participants: Adult primary care patients with risky drug use range 4–26 on the WHO ASSIST self-administered on tablet computers: 65 patients (32 intervention, 33 control); 51 (78%) completed follow-up; mean age 30.8 years; 59% male; 94% Latino. Interventions and measures: Intervention patients received: (1) brief (typically 3–4 min) clinician advice to quit/reduce their risky drug use, (2) video doctor message reinforcing the clinician’s advice, (3) health education booklet, and (4) up to two 20–30 min follow-up telephone drug use reduction coaching sessions. Control patients received usual care and cancer screening information. Primary outcome was reduction in the number of days of drug use in the past 30 days of the highest scoring drug (HSD) measured on the ASSIST, from baseline to 3-month follow-up.

**Results:** Intervention patients reduced their past month HSD use by 4.5 more days than controls (*p* < .042, 95% CI 0.2, 8.7) by 3-month follow-up in an intent-to-treat linear regression analysis. Similar significant results were found using a complete sample regression analysis: 5.2 days (*p* < .03, 95% CI 0.5, 9.9).

**Conclusions:** Findings further support the efficacy of QUIT in reducing risky drug use.

## A34 Web-based intervention for people with harmful use or dependence of alcohol

### Magnus Johansson^1^, Christina Sinadinovic^2^, Anne H. Berman^2^, Ulric Hermansson^2^, Sven Andreasson^1^

#### ^1^Department of Public Health Sciences, Karolinska Institute, Stockholm, Sweden; ^2^Department of Clinical Neuroscience, Center for Psychiatry Research, Karolinska Institutet, Stockholm, Sweden

##### **Correspondence:** Magnus Johansson - magnus.johansson.1@ki.se

*Addiction Science & Clinical Practice* 2017, **12(Suppl 1)**: A34

**Background:** Few harmful or dependent drinkers ever seek professional help. This is largely due to stigma. Harmful drinkers are ashamed of their problem and of going to a clinic. Many people prefer to use the internet to get information about alcohol over asking a doctor or friend. Web-based interventions for alcohol-problems reach individuals who to a lesser extent come into contact with traditional addiction services. The interventions have shown small to moderate effects in reducing alcohol consumption. Few studies have focused on alcohol dependent users or the additional effect of guidance.

**Materials and Methods:** A 3-arm randomized controlled trial was conducted at a well-established Swedish website aimed at the general public. New users from March 2015 to March 2017, with alcohol harmful use or dependence, were offered to participate. After completing baseline questionnaires, all participants answered a survey about reasons for and preferences regarding web-based interventions. After submitting the survey they were randomized to one of three forms of support: (1) information, (2) program as self-help or (3) program with on-line contact with a therapist. Participants were blinded to what kind of support the other groups received. The 8 module program consists of information-texts, videos and exercises based on Cognitive Behavioral Therapy and Motivational Interviewing.

**Results:** Of 1175 participants with a mean age 45 years (SD = 13), 56% were women and 89% alcohol dependent (ICD). In the 7 days prior to study inclusion, participants consumed an average of 26 (sd = 17) drinks and their mean AUDIT score was 22 (SD = 6). Further, 37% showed symptoms of generalized anxiety (GAD-7) and 43% of depression (MADRS-S). Participants were more ready to reduce their drinking (m = 8.4; SD = 1.9, on a 0–10 VAS-scale) than to stop (m = 8.4; SD = 1.9). The most endorsed reasons for using web-based intervention were anonymity and having access to intervention at any time. The most endorsed features were assessment feedback and online contact with a therapist. Data collection from the 3-month follow-up will be completed in July, 2017.

**Discussion:** Anonymity and access might be important reasons for choosing web-based treatment. The results from the randomized study will add knowledge of the effectiveness of web-based interventions, with or without guidance.

## A35 Examination of referral to treatment as part of the brief intervention model

### Megan A. O’Grady^1^, Sandeep Kapoor^2^, Cherine Akkari^1^, Camila Bernal^1^, Kristen Pappacena^1^, Jeanne Morley^2^, Mark Auerbach^3^, Charles J. Neighbors^1^, Nancy Kwon^3^, Joseph Conigliaro^2^, Jon Morgenstern^4^

#### ^1^Division of Health Services Research, The National Center on Addiction and Substance Abuse, New York, NY, USA; ^2^Department of Internal Medicine, Emergency Medicine, and Psychiatry, Northwell Health, Great Neck, NY, USA; ^3^Departent of Emergency Medicine, Northwell Health, Great Neck, NY, USA; ^4^Department of Psychiatry, Northwell Health, Great Neck, NY, USA

##### **Correspondence:** Megan A. O’Grady - mogrady@centeronaddiction.org

*Addiction Science & Clinical Practice* 2017, **12(Suppl 1)**: A35

**Background:** Screening, brief intervention, and referral to treatment (SBIRT) models in medical settings often rely on referrals to community treatment programs when patients with potential substance use disorder are identified. Referrals have been studied much less than the screening and brief intervention components of the SBIRT model. In the available referral research there are mixed findings, with some studies showing that SBIRT increases treatment entry; others finding no relationship. Previous research also suggests that patient characteristics may be important in understanding the SBIRT referral process. As part of an evaluation of a large-scale SBIRT program, we examined the proportion of referred patients who entered treatment, as well as patient-level predictors of treatment entry.

**Materials and methods:** SBIRT was implemented in four emergency departments (ED) and 4 primary care practices in a large health system. Over 6000 patients screened positive on the AUDIT or DAST-10 during the first 3.5 years of the program; 1091 (mostly ED patients [90%]) received a referral. About 40% consented to participation in the referral evaluation by allowing us to link program evaluation data with the New York State Client Data System treatment registry (n = 407). Program evaluation data included demographics, screening scores, drug and alcohol use patterns, and health and psychosocial information. State data included date of treatment entry and level of care entered.

**Results:** Fifty-five percent of participants drank daily. During the past 30 days, 21% used marijuana and cocaine and 16% used opiates. Twenty-five percent (107) of participants (99% from ED) entered treatment within 90 days of SBIRT (30% same day; 20% 1–7 days; 25% 8–30 days; 25% 31–90 days). The majority entered detox (79%); the remaining entered inpatient (12%) or outpatient (9%) programs. Multivariate logistic regression analyses showed that having employment and trauma history decreased the likelihood of entering treatment within 90 days.

**Conclusions:** We found that a quarter of patients receiving referral as part of SBIRT entered treatment and identified important patient-level referral predictors. SBIRT programs should continue to examine ways to increase treatment entry among those most in need. Patients with trauma history may benefit from trauma-informed SBIRT models.

## A36 The long view of meta-analysis: testing technical, relational, and conditional process models in brief motivational intervention

### Molly Magill^1^, Timothy R. Apodaca^2^, Brian Borsari^3^, Jacques Gaume^4^, Ariel Hoadley^5^, J. Scott Tonigan^6^, Theresa Moyers^6^

#### ^1^Center for Alcohol and Addiction Studies, Brown University, Providence, RI, USA; ^2^Children’s Mercy Kansas City, University of Missouri-Kansas City School of Medicine, Kansas City, MO, USA; ^3^San Francisco Veterans Affairs Health System and Department of Psychiatry, University of San Francisco, San Francisco, CA, USA; ^4^Lausanne University Hospital, Lausanne, Switzerland; ^5^School of Public Health, Brown University, Providence, RI, USA; ^6^Center on Alcoholism, Substance Abuse, and Addictions, University of New Mexico, Albuquerque, NM, USA

##### **Correspondence:** Molly Magill - molly_magill@brown.edu

*Addiction Science & Clinical Practice* 2017, **12(Suppl 1)**: A36

**Background:** In the present meta-analysis, we test the technical and relational hypotheses of Motivational Interviewing (MI) efficacy. We also propose an a priori conditional process model where heterogeneity of technical path effect sizes should be explained by interpersonal/relational (i.e., empathy, MI Spirit) and intrapersonal (i.e., client treatment seeking status) moderators.

**Materials and methods:** A systematic review identified k = 58 reports, describing 36 primary studies and 40 effect sizes (N = 3025 participants). Statistical methods calculated the inverse variance-weighted pooled correlation coefficient for the therapist to client and the client to outcome paths across multiple target behaviors (i.e., alcohol use, other drug use, other behavior change).

**Results:** Therapist MI-consistent skills were correlated with more client change talk (r = .55, *p* < .001) as well as more sustain talk (r = .40, *p* < .001). MI-inconsistent skills were correlated with more sustain talk (r = .16, *p* < .001), but not less change talk. When these measures were combined, as recommended in the Motivational Interviewing Skill Code, the overall technical hypothesis was supported. Specifically, proportion MI consistency was related with higher proportion change talk (r = .11, *p* = .004) and higher proportion change talk was related with reductions in risk behavior at follow up (r = −.18, *p* < .001). When tested as two independent effects, however, client change talk was not significant, but sustain talk was positively associated with worse outcomes (r = .20, *p* < .001). Finally, the relational hypothesis was not supported, but heterogeneity in technical hypothesis paths was partially explained by the a priori moderators of interest.

**Conclusions:** This meta-analysis provides additional support for the technical hypothesis of MI efficacy.

## A37 Financial incentives for alcohol brief interventions in primary care in Scotland

### Niamh M. Fitzgerald^1^, Lisa Schölin^2^, Amy J. O’Donnell^3^

#### ^1^Institute for Social Marketing, UK Centre for Tobacco and Alcohol Studies, University of Stirling, Stirling, Scotland, UK; ^2^World Health Organization Regional Office for Europe; formerly Institute for Social Marketing, University of Stirling, Stirling, Scotland, UK; ^3^Institute of Health and Society, Newcastle University, Newcastle upon Tyne, UK

##### **Correspondence:** Niamh M. Fitzgerald - niamh.fitzgerald@stir.ac.uk

*Addiction Science & Clinical Practice* 2017, **12(Suppl 1)**: A37

**Background:** This study aimed to examine the evidence for financial incentives for screening and brief intervention for alcohol (SBI) in primary care and to explore the remuneration systems established in Scotland under the Scottish Government’s national SBI programme established in 2008.

**Materials and methods:** A rapid systematic literature review on the design and impact of financial incentives on the delivery of SBI in primary care, using PubMed; analysis of documentation and data regarding remuneration systems in three local areas in Scotland; in-depth semi-structured interviews with 5 key local and national stakeholders on the design and impact of remuneration models in Scotland.

**Results:** From the 235 titles identified in the systematic search, ten underwent full text review and four met inclusion criteria. Evidence in this area: is scarce, particularly in relation to systems implemented in routine practice; provides mixed evidence of impact on patient and provider outcomes; and does not indicate an optimum level of incentive. The three local remuneration models varied considerably in structure and rates of payment over time and in different areas: one provided core funding for community nursing; one made a single payment for SBI (only where patients screened as needing intervention); and two paid separate incentives for screening and for brief interventions following screening. No firm conclusions could be drawn about optimal models or levels of payment or the impact of changes over time. The ratio of brief interventions to screenings delivered also varied widely. Interviewees disagreed on whether incentives led to increased SBI delivery but suggested that the remuneration contracts enabled training and monitoring of delivery to be mandated. Distortions such as misrepresentation or gaming by claimants were not thought to be widespread.

**Conclusions:** The establishment of 14 different local SBI remuneration systems in Scotland provided several opportunities for evaluation of natural experiments which have largely been missed. This study raises the possibility that financial incentives operate as interventions in a complex system, rather than just incentivizing SBI delivery. Their structure, targets, and value are likely to have important implications, which ought to be studied carefully as part of, and to inform, future implementation initiatives.

## A38 Acceptability and usability of a tablet-based device for substance use and physical activity screening in primary care

### Nicolas Bertholet^1^, Angéline Adam^1^, John A. Cunningham^2^, Jean-Bernard Daeppen^1^

#### ^1^Alcohol Treatment Center, Department of Community Medicine and Health, Lausanne University Hospital, Lausanne, Switzerland; ^2^Centre for Addiction and Mental Health, Toronto, Ontario, Canada

##### **Correspondence:** Nicolas Bertholet - Nicolas.Bertholet@chuv.ch

*Addiction Science & Clinical Practice* 2017, **12(Suppl 1)**: A38

**Background:** In primary care practices, electronic screening can potentially overcome implementation barriers and help clinicians. Acceptability and usability are key elements in successful implementation.

**Materials and methods:** With the help of product designers, we developed a tablet-based device for primary care waiting rooms. The device was designed to inspire ease and comfort and the tablet program was carefully designed to maximize ergonomics. It comprises screening for tobacco, alcohol, illicit drugs, prescription drug use and physical activity. A summary of the screening results with emoticons is then presented to patients. Patients who screen positive have the option to answer additional questions on alcohol use and to receive an electronic brief intervention. In February 2017, the device was piloted in 4 different primary care practices in suburban and rural Switzerland. The aim was to assess acceptability and usability of the device. On random half-days, patients in the waiting room were encouraged by a research assistant to use the device and asked to complete a satisfaction questionnaire.

**Results:** During the evaluation period and while the research assistant was present, 280 patients attended the practices and 99 (35.4%) used the device. Of them, 82 (82.3%) completed the satisfaction questionnaire. Mean (SD) age was 49.9 (16.9), 54.5% were female; 94% considered the device easy to use (“agree” or “strongly agree”), 93% considered the questions easy to understand, 79% considered their friends would be willing to use the device, while 8% reported that answering the questions made them uncomfortable, and 12% that they would prefer if the primary care physician asked the questions. Most considered “useful” or “very useful” to be asked about their tobacco (92%), alcohol (92%), drug (99%), prescription drug use (91%) and physical activity (87%).

**Conclusions:** Among patients who used the device, its acceptability and usability were good. The developed device appears easy to use and patients generally perceived being asked about substance use and physical activity as useful. Nevertheless, the proportion of patients accessing the device remained limited.

## A39 AUDIT-linked Brief Intervention delivered by paramedics in Chilean primary care: a pragmatic randomized controlled trial

### Nicolas Barticevic^1^, Soledad Zuzulich^2^, Fernando Poblete^3^, Pablo Norambuena^4^

#### ^1^Department of Family Medicina, Pontificia Universidad Católica de Chile, Santiago de Chile, Región Metropolitana, Chile; ^2^Nursing School, Pontificia Universidad Católica de Chile, Santiago de Chile, Región Metropolitana, Chile; ^3^Public Health Department, Pontificia Universidad Católica de Chile, Santiago de Chile, Región Metropolitana, Chile; ^4^Mental Health Department, Ministry of Health, Chile

##### **Correspondence:** Nicolas Barticevic - nicolas.barticevic@gmail.com

*Addiction Science & Clinical Practice* 2017, **12(Suppl 1)**: A39

**Background:** In Chile, alcohol use is the leading cause of years lost due to disability. To address this issue, the Health Ministry has implemented a national program on Brief Interventions (BI) for Risky Alcohol use, in which Paramedics are the main BI providers. The objective of this work is to study the effectiveness of non-professional delivered BIs as they occur in the real world in Chilean primary care.

**Materials and Methods:** A multi-center randomized open-label controlled trial was conducted in five primary care centers in Santiago de Chile. A total of 3247 people aged 18–45 were screened for moderate-risk alcohol use according to AUDIT (Alcohol Use Disorders Identification Test), and 343 participants were randomized. The paramedic-delivered BI (n = 174) was compared to an informative pamphlet (n = 169). The outcome measure was the AUDIT score at baseline and six months follow-up.

**Results:** From the total people screened, 11% were at moderate risk for alcohol use (AUDIT 8–15), and 2% at high risk (AUDIT over 15). Recruited participants had a mean age of 29 years, 57% were male, and the average AUDIT score was 10.5 (SD 2.6), which did not differ between groups. Changes to the “real practice” conditions had to be made to permit full protocol implementation. Lack of time, competition with other tasks, and lack of space were some of the problems for screening procedures and delivery of BI. 58 paramedics were trained on AUDIT and BI delivery, and 32 reached the standards to participate in the study. Additional training was needed to ensure proper AUDIT administration and BI structure. Only 10 paramedics finally participated in the study, mainly due to administrative constraints. To date, 120 participants have completed follow-up. The mean AUDIT score at follow-up is 6.2 for all participants, with no significant differences observable between groups so far.

**Conclusions:** Paramedics can implement AUDIT linked BIs, but they need special training and accommodation to integrate it into their practice and tasks. Both groups had lower AUDIT scores (by 4 points), but completion of follow-up is necessary to determinate if there is an effect attributable to BI.

## A40 The integration of SBIRT into social work curricula at a university setting

### Paul Sacco^1^, Laura Ting^2^, Michele Beaulieu^1^

#### ^1^School of Social Work, University of Maryland-Baltimore, Baltimore, MD, USA; ^2^University of Maryland-Baltimore County, Baltimore, MD, USA

##### **Correspondence:** Paul Sacco - PSACCO@ssw.umaryland.edu

*Addiction Science & Clinical Practice* 2017, **12(Suppl 1)**: A40

**Background:** Presenters will review their SAMHSA-developed SBIRT curriculum focused on at-risk drinking, tobacco use and other drug use in social work (MSW) education. The curriculum was implemented through two methods: a standalone SBIRT (15 h, 1 credit) course and a hybrid foundation course infusion model.

**Materials and methods:** Standalone course students (n = 83) received in-person didactic instruction using an adapted version of the SAMHSA-SBIRT curriculum with role-plays, demonstration videos, and values clarification exercises. All foundation first year MSW students (n = 728) completed the hybrid infusion model combining 6 h of online and in-class training, including role-play exercises, videos and didactic instruction. All trainees completed satisfaction surveys and self-report scales assessing SBIRT knowledge, attitudes, and behaviors pre-intervention, post-intervention (30 day) and 6-months post-intervention. Trainees in the standalone course were also evaluated through videotaped standardized patient interviews (pre- and immediate post-test) assessing their SBIRT skills and coded by trained staff using a standardized rating scale. Qualitative interviews focused on students’ perceptions of SBIRT and their use of SBIRT in practice.

**Results:** Overall, the social work SBIRT project has reached comparable or greater numbers of health professionals than 2013–2016 SAMHSA grantees; levels of trainee satisfaction were comparable with similar programs. Data from standalone coursework suggests confidence, knowledge and behavior change increased due to training, but leveled off at 6-months post-training (Sacco et al., in press). Qualitative interviews indicate trainees have difficulties implementing SBIRT due to lack of clarity about whether it was their role (or any social worker’s job) to conduct screening and brief intervention. Trainees also felt uncomfortable making recommendations to agency administrators to implement SBIRT given their student role. Additional structural barriers prevent SBIRT implementation as agency funding mandates require the use of other, specific assessment tools, or limit substance use screening to addiction counselors.

**Conclusions:** Our SAMHSA project has been successful in training significant numbers of social work students. Student satisfaction has been high and training outcomes suggest that the training is effective. Uptake of SBIRT by social work interns in practice has been limited. System-level factors and social worker perceptions about scope of practice may be barriers to implementation.

## A41 Use of SMS texts for facilitating access to online alcohol interventions: a feasibility study

### Paul George Wallace^1^, Matthew Andrews^2^, Kate Daley^3^, Don Shenker^4^, Louise Gallagher^5^, Rod Watson^6^, Tim Weaver^7^, Amy O’Donnell^8^

#### ^1^South London Health Innovation Network, London, UK; ^2^Safe Sociable London Partnership, London, UK; ^3^South London Health Innovation Network, London, UK; ^4^Alcohol Health Network, London, UK; ^5^Department Public Health, Royal Borough of Kingston, London, UK; ^6^South London Health Innovation Network, London, UK; ^7^Middlesex University, London, UK; ^8^University of Newcastle, Newcastle, UK

##### **Correspondence:** Paul George Wallace - paul.wallace@nihr.ac.uk

*Addiction Science & Clinical Practice* 2017, **12(Suppl 1)**: A41

**Background:** Facilitated access to online alcohol intervention can have a significant impact on reducing alcohol misuse and ill-health and offers a potentially cost effective alternative to face to face intervention. SMS text messaging offers a novel means to implement facilitated access from general practice and this project was designed to test its feasibility and potential utility.

**Materials and methods:** A London practice used iPLATO software to send campaign and reminder SMS texts to 406 patients who had previously completed the AUDIT-C. The texts included a short message—“Healthier drinking choices? Take the alcohol health check http://e-drink-check.kingston.gov.uk/ref/p1c1”. The online package accessed via the link enabled the patient to respond online to the AUDIT questions and to be categorized as low risk, increasing risk, high risk or possibly dependent. Respondents were then provided with brief advice and health-risk feedback on their score, based on motivational interviewing techniques, designed to increase self-awareness and provide techniques to address the issue and motivate health improvement.

**Results:** Overall 45/406 (11.1%) accessed the website within 4 days of text (8th–12th March), most on the first day. 43 respondents provided their gender—47% were women and 53% were men. Of the 38 who provided data, 25 (66%) were managers or professionals. 45 started the AUDIT, 41 (10.1%) completed the AUDIT-C, and 35 (8.6%) the full AUDIT. Of those who completed AUDIT-C, 36 (87.8%) scored positive (5 +). 3 registered for drink diary. Of the 35 completing the AUDIT, 10 were low risk (28.6%), 16 were increasing risk (45.7%), 5 were high risk (14.3%) and 4 (11.4%) were possibly dependent.

**Conclusions:** This study suggests that it may well be feasible to use SMS messaging as a means to facilitate patient access to on line screening and brief intervention packages. Such an approach offers real promise for substantially increasing the delivery of screening and brief intervention in primary care, but larger studies will be required to establish the cost effectiveness of this approach compared with traditional face to face delivery.

## A42 Feasibility and effectiveness of a specialized brief intervention for hazardous drinkers in an emergency department

### Pol Bruguera^1^, Clara Oliveras^2^, Carolina Gavotti^3^, Pablo Barrio^3^, Fleur Braddick^3^, Hugo López-Pelayo^3^, Laia Miquel^3^, Montse Suárez^4^, Carla Bruguera^5^, Lídia Segura^5^, Joan Colom^5^, Antoni Gual^3^

#### ^1^Grup de Recerca, Addiccions Clínic (GRAC), Addictive Behaviours Unit, Psychiatry Department, Hospital Clínic de Barcelona, Barcelona, Spain; ^2^Psychiatry Department, Hospital Clínic de Barcelona, Barcelona, Spain; ^3^Grup de Recerca Addiccions Clínic (GRAC), Addictive Behaviours Unit, Psychiatry department, Hospital Clínic de Barcelona, Barcelona, Spain; ^4^Emergency Department, Hospital Clínic de Barcelona, Barcelona, Spain; ^5^Program on Substance Use, Public Health Agency, Government of Catalonia, Barcelona, Spain

##### **Correspondence:** Antoni Gual and Pol Bruguera - tgual@clinic.cat and pbruguer@clinic.cat

*Addiction Science & Clinical Practice* 2017, **12(Suppl 1)**: A42

**Introduction:** Screening, brief intervention, and referral to treatment (SBIRT) programs have been developed, evaluated and shown to be effective, particularly in primary care and general practice. Nevertheless, effectiveness of SBIRT in emergency departments (EDs) has not been clearly established. We aimed to evaluate the feasibility and efficacy of an SBIRT program conducted by psychiatrists specialized in addictive disorders and motivational interviewing techniques in the ED of a tertiary hospital.

**Materials and methods:** We conducted a randomized controlled trial to study the feasibility and efficacy of an SBIRT programme for hazardous drinkers presenting in an ED. All patients older than 18 years attending the ED were potentially eligible. Cognitively impaired or medically unstable patients were excluded, as were patients seeking treatment for alcohol use. Patients were randomized to two groups, with the control group receiving two leaflets—one regarding alcohol use, and the other giving information about the study protocol. The intervention group received the same leaflets as well as a brief motivational intervention on alcohol use and, where appropriate, a referral to specialized treatment. The primary outcomes were the proportion of hazardous drinkers measured by AUDIT-C scale and the proportion of patients attending specialized treatment at 1.5 and 4.5 months.

**Results:** Of 3027 patients attending the ED, 2044 (67%) were potentially eligible to participate, 247 (12%) screened positive for hazardous drinking, and 200 agreed to participate. 72% of the participating sample were men, and the mean age was 43 years. Follow-up rate was 78%. At 1.5 months, the intervention group showed greater reductions in alcohol consumption, and fewer patients continuing with hazardous alcohol use (26.4 vs 48.1%, *p* = 0.0053). The SBIRT program also increased the probability of attending specialized treatment, compared to the control condition (19.4 vs 6.1%, *p* = 0.0119).

**Conclusion:** The SBIRT program in the ED was found to be feasible and effective in identifying hazardous drinkers, reducing hazardous alcohol use and increasing treatment for alcohol problems.

## A43 A dissemination model for delivering SBI in high schools

### Richard L. Brown^1^, Julie Whelan Capell^2^, D. Paul Moberg^3^, Julie Maslowsky^4^, Laura A. Saunders^1^

#### ^1^Department of Family Medicine and Community Health, University of Wisconsin School of Medicine and Public Health, Madison, WI, USA; ^2^JulieINK, LLC, Milwaukee, WI, USA; ^3^Population Health Institute, University of Wisconsin-Madison, Madison, WI, USA; ^4^Department of Kinesiology and Health Education, University of Texas-Austin, Austin TX, USA

##### **Correspondence:** Richard L. Brown - rlbrown@wisc.edu

*Addiction Science & Clinical Practice* 2017, **12(Suppl 1)**: A43

**Background:** Alcohol and drug screening and brief intervention (SBI) prolongs abstinence and reduces substance use for adolescents. Numerous barriers to delivering SBI in healthcare settings have been delineated. Dissemination models are needed so that all adolescents can receive SBI.

**Materials and methods:** Ten high schools in Southeastern Wisconsin, USA, were recruited as sites for universal SBI administration to all ninth- or tenth-graders. School administrators agreed to use opt-out recruiting with students and parents. They expressed a preference for SBI administration by non-school personnel, as school staff did not have time, and students would reveal more accurate information. In January, 2016, eight college seniors were trained to serve as health coaches and administer SBI. Between January and May, 2016, they delivered SBI at the high schools. Most sessions lasted about 15 min. Each student initially completed screening and brief assessment questionnaires via computer. The computer immediately printed out a summary of the student’s responses. The coach reviewed the computer printout and administered motivational interventions aimed to prolong abstinence or reduce use. Each student returned to the computer at the end of the session and anonymously answered several questions about their experience with the health coach and its impacts.

**Results:** Over 95% of students in the participating grades received SBI. About two-thirds of the 2525 students who received SBI were freshmen, and one-third were sophomores. Self-reported prevalence of past-year substance use was 18% for alcohol, 10% for marijuana, and 3% for other drugs. Over 95% of the students reported comfort talking with their health coach and trusted that the information they revealed would remain confidential, as coaches promised. After the SBI session, 87% of the past-year drinkers, 75% of the past-year marijuana users, and 80% of the past-year users of other drugs reported stronger intention to reduce their substance use in the next month. Over 94% of the abstinent students reported stronger intention to continue abstinence over the next year.

**Conclusions:** SBI administration by non-school staff holds promise as a method of delivering universal SBI to high school students.

## A44 Using the alcohol single-item screening question in the emergency department for screening and risk-stratification

### Ryan P. McCormack^1^, Joy Scheidell^2^, Mirelis Gonzalez^1^

#### ^1^Department of Emergency Medicine, NYU School of Medicine, New York, NY, USA; ^2^Department of Population Health, NYU School of Medicine, New York, NY, USA

##### **Correspondence:** Ryan P. McCormack - Ryan.McCormack@nyumc.org

*Addiction Science & Clinical Practice* 2017, **12(Suppl 1)**: A44

**Background:** The adoption of screening and intervention for unhealthy alcohol use in emergency departments (EDs) remains extremely limited. Streamlining the assessment would be more acceptable to staff, who cite time and competing priorities as barriers. The single-item screening question (SISQ) to identify unhealthy alcohol use may also provide information on severity to inform the brief intervention (Saitz et al. 2009, 2010, 2014). We assessed the SISQ accuracy for identifying which ED patients with unhealthy alcohol use should receive a brief intervention versus brief advice.

**Materials and methods:** Non-clinician health coaches conducted brief health surveys on a non-targeted, random sample of English- or Spanish-speaking adult patients at an urban, public hospital during the hours of 8 a.m. and 12 a.m. on all days of the week. Patients were asked the SISQ, “How many times in the past 12 months have you had [X] or more alcoholic drinks in a day?” (where X is 5 for men and 4 for women, and any use is considered positive). Patients with positive responses were asked to use the following frequency options: “Never,” “Less than monthly,” “Monthly,” “Weekly,” “Daily or almost daily.” These options were preferred to numerical frequencies during piloting. All patients who screened positive (only) were asked the 10-item Alcohol Use Identification Test (AUDIT). We compared the SISQ responses to the AUDIT dichotomized at 15; AUDIT scores of 0–15 receive education and advice; scores of >15 receive brief intervention and possible referral.

**Results:** Among 1220 patients who screened positive for unhealthy alcohol use via the SISQ, 882 (72.3%) had an AUDIT score of 0–15 and 338 (27.7%) had a score >15. The sensitivity and specificity of the SISQ in detecting an AUDIT score of >15 is 89.3% and 74.0%, respectively, when the SISQ frequency of heavy drinking is dichotomized at less than or equal to monthly versus weekly or more.

**Conclusion:** In this sample of ED patients with unhealthy alcohol use, the reported SISQ frequency of heavy drinking can be used as an indicator of severity to differentiate between patients who should receive brief advice versus a more substantive intervention.

## A45 Preparing the future workforce: development and field test evaluation of adolescent SBI simulation training

### Weiwei Liu^1^, Tracy L. McPherson^1^, Sabrina Bauroth^1^, Cyrille Adam^2^, Dawn L. Lindsay^3^, Piper Lincoln^3^, Holly Hagle^4^

#### ^1^Public Health, NORC at the University of Chicago, Bethesda, MD, USA; ^2^Research and Development, Kognito, New York, NY, USA; ^3^Research and Evaluation, Institute for Research, Education and Training in Addiction, Pittsburgh, PA, USA; ^4^Education and Training, Institute for Research, Education and Training in Addiction, Pittsburgh, PA, USA

##### **Correspondence:** Tracy McPherson - McPherson-Tracy@norc.org

*Addiction Science & Clinical Practice* 2017, **12(Suppl 1)**: A45

**Background:** Nursing and social work programs from across the U.S. were recruited to participate in a learning collaborative to integrate adolescent SBI training into curriculum. NORC partnered with the American Association of Colleges of Nursing, Council on Social Work Education, Center for Clinical Social Work, IRETA, Kognito, and subject matter experts (SMEs) to develop and evaluate an adolescent alcohol and marijuana SBI online simulation training program. The program aims to prepare students, educators, and practitioners to conduct Brief Negotiated Interviews using Motivational Interviewing (MI) strategies. This study aimed to evaluate the effectiveness of the training on key outcomes including students’ knowledge; attitudes toward working with patients/clients who use alcohol; readiness, confidence, and competence; and adolescent SBI skills.

**Materials and methods:** The SBI with Adolescents simulation training program was developed in collaboration with partners and SMEs, then beta tested and usability tested. Following development and testing, schools of nursing and social work (n = 11) were recruited from the learning collaborative to participate in the field test evaluation. Among the 1390 nursing and social students who participated, 593 (42.7%) accessed the training; 797 (57.3%) did not, facilitating the comparison between trained and untrained students on key outcomes. Students completed a pretest and two posttest surveys online, approximately 30 days apart. OLS regression was used to compare trained and untrained students at the two post-tests, while controlling for pretest. Longitudinal growth modeling was used to assess the effect of the simulation training on the starting point and change of a given outcome.

**Results:** Comparisons of demographic characteristics between trained and untrained students showed no differences by gender and age. The simulation training was effective in improving a number of key outcomes. Results showed a small to medium standardized effect size, and students who received the training showed sharper growth in their attitudes, confidence, competency, and readiness to implement SBI in the field at the posttest.

**Conclusions:** The findings suggest that online simulation training can positively impact student outcomes and shows great promise as a method for preparing students to conduct brief interventions using MI skills with adolescents using alcohol and marijuana.

## A46 Treatment of alcohol dependence in primary care compared to specialist care: a randomised controlled trial

### Sara Wallhed Finn^1^, Anders Hammarberg^2^, Sven Andréasson^1^

#### ^1^Department of Public Health Sciences, Karolinska Institutet, Centre for Psychiatry Research, Stockholm Health Care Services, Stockholm, Sweden; ^2^Department of Clinical neurosciences, Karolinska Institutet, Centre for Psychiatry Research, Stockholm Health Care Services, Stockholm, Sweden

##### **Correspondence:** Sara Wallhed Finn - sara.wallhed-finn@ki.se

*Addiction Science & Clinical Practice* 2017, **12(Suppl 1)**: A46

**Background:** A minority of all individuals with alcohol dependence seek treatment. A possible way to reduce this treatment gap is to offer treatment in primary care. Most treatment studies in primary care have included individuals with hazardous consumption, and alcohol dependence has been studied to a lesser extent. There is a need to develop brief and effective treatment models that are feasible to implement. We have developed a stepped care model for treatment of hazardous drinking and alcohol dependence in primary care, “the 15-method”. The model consists of three steps: (1) identification of problem drinking and brief advice, (2) assessment, with feedback, and (3) four brief sessions based on CBT and motivational interviewing. These sessions can be combined with pharmacological treatment. In this trial steps (2) and (3) are studied. Objective: To investigate if treatment for alcohol dependence, using a stepped care model, in primary care is as effective as specialist addiction care.

**Materials and methods:** Randomized controlled non-inferiority trial, between groups parallel design, not blinded. The non-inferiority limit was set to 50 g of alcohol per week. 288 adults fulfilling ICD-10 criteria for alcohol dependence were randomized to treatment in primary care (n = 144) or specialist care (n = 144). General practitioners at 12 primary care centers received one day of training in the model. Primary outcome was change in weekly alcohol consumption at twelve months follow up compared to baseline, as measured with Time Line Follow Back. Secondary outcomes were heavy drinking days, severity of dependence, consequences of drinking, psychological health, quality of life, satisfaction with treatment and biomarkers.

**Results:** Preliminary results of the intention-to-treat analysis (n = 231) confirms non-inferiority for the primary outcome at twelve months follow up. Weekly alcohol consumption in primary care (n = 111) was 9.7 grams higher compared to specialist care (n = 120), (95% CI −30.4 to 49.7), *p* = 0.64.

**Conclusions:** A stepped care model is a promising approach for treatment of alcohol dependence in primary care. This may be a way to broaden the base of treatment for alcohol dependence, reducing the current treatment gap.

## A47 Barriers to and facilitators of integrating adolescent SBIRT training in nursing, social work and inter-professional education

### Sarah E. King^1^, Dawn L. Lindsay^2^, Tracy L. McPherson^1^, Rachael Vargo^2^, Brayden N. Kameg^3^, Holly Hagle^4^

#### ^1^Public Health Department, NORC at the University of Chicago, Bethesda, MD, USA; ^2^Research and Evaluation, Institute for Research, Education and Training in Addiction, Pittsburgh, PA, USA; ^3^Department of Health and Community Systems, University of Pittsburgh School of Nursing, Pittsburgh, PA, USA; ^4^Training and Education, Institute for Research, Education and Training in Addiction, Pittsburgh, PA, USA

##### **Correspondence:** Sarah E. King - king-sarah@norc.org

*Addiction Science & Clinical Practice* 2017, **12(Suppl 1)**: A47

**Background:** Despite the support for screening and brief intervention to identify alcohol and related problems in medical and behavioral health settings, this skill is rarely integrated into the professional education of future practitioners. NORC, in partnership with the Council on Social Work Education and the American Association of Colleges of Nursing, led a multi-year learning collaborative to infuse adolescent SBIRT curriculum in schools of social work and nursing.

**Materials and methods:** NORC partnered with IRETA on secondary analysis utilizing the Consolidated Framework for Implementation Research (CFIR) to conceptualize barriers and facilitators to the implementation of adolescent SBIRT curriculum into undergraduate and graduate nursing and social work programs. Data from implementation progress reports, learning collaborative calls, and implementation calls with individual schools working on infusing SBIRT curricula were reviewed by two raters and categorized according to the CFIR model.

**Results:** Across study activities, barriers and facilitators of curriculum implementation appeared in several key stakeholder groups: students, educators (including faculty, field supervisors, nurse preceptors), and program administrators. A number of key elements specific to the CFIR components of Inner Setting, Characteristics of Individuals, and Process emerged such as stakeholder and leadership buy-in; previous exposure to SBIRT; comfortability with substance use/mental health issues; familiarity with technology; utilization of program liaisons/champions; curriculum and course adaptability; and training of field supervisors and preceptors.

**Conclusions:** Pre-service education is a critical component to preparing the medical and behavioral workforce to implement SBIRT in a range of settings. The CFIR model identified barriers to and facilitators of curriculum integration found in nursing, social work, and inter-professional education. Findings from this study can help inform approaches to the integration of SBIRT education taken by educators and program leaders in academic institutions around the globe. Moreover, the findings can be used by professional associations who provide guidance on educational standards and curriculum infusion.

## A48 SBIRT in an interprofessional context for healthcare students and professionals

### Shauna P. Acquavita^1^, Ruth Anne Van Loon^2^, Rachel Smith^2^, Bonnie J. Brehm^3^, Tiffiny Diers^4^, Karissa Kim^5^, Andrea Barker^2^

#### ^1^School of Social Work, College of Allied Health Sciences, University of Cincinnati, Cincinnati, OH, USA; ^2^University of Cincinnati, School of Social Work, Cincinnati, OH, USA; ^3^College of Nursing, University of Cincinnati, Cincinnati, OH, USA; ^4^College of Medicine, University of Cincinnati, Cincinnati, OH, USA; ^5^James L. Winkle College of Pharmacy, University of Cincinnati, Cincinnati, OH, USA

##### **Correspondence:** Shauna P. Acquavita - acquavsa@ucmail.uc.edu

*Addiction Science & Clinical Practice* 2017, **12(Suppl 1)**: A48

**Background:** Students across the healthcare professions need SBIRT knowledge and skills to be prepared to work with patients in a variety of settings. An interprofessional SBIRT course for students in medicine, nursing, pharmacy, and social work was developed to provide this education and to prepare students for collaborative practice.

**Materials and methods:** SBIRT education was presented in a hybrid course. In the first half, students completed online modules on substance use disorders (SUD) and had SBIRT virtual simulations. The mid-term exam was a standardized patient experience where interprofessional teams administered SBIRT. Finally, clinical experiences in community agencies and the university’s medical center allowed interprofessional teams to implement SBIRT with patients/clients. Quantitative and qualitative data provided feedback on course content and experiences.

**Results:** Prior to the course, most students (n = 43) had ten hours or less of training in SUD (81%), course content on motivational interviewing (MI) (95%), or experience using MI with clients (58%). There were significant improvement in scores for competency in SBIRT (Measured by University of Pittsburgh’s SBIRT Medical and Residency Training Survey), from pre to post course (*p* < .0005). Perceived confidence and preparedness (*p* < .0005) for conducting a SBI improved after completing a SBI virtual simulation. Qualitative data included “The experiences gave me an excellent opportunity to apply SBIRT in a real setting… and allowed me to work collaboratively with other professions that may play a role in the impact alcohol and drugs has on one’s health. (Social Work)”; “If other members of the healthcare team are also trained in SBRT skills then the chances of a patient receiving the proper screening and intervention would be greatly increased…In the future, I plan to share my SBRT training…” (Medicine).”

**Conclusions:** An interprofessional hybrid course is an effective method for providing education about SUD and interventions and for teaching SBIRT skills, topics often missing from disciplinary curricula. The hybrid model meets the challenges of scheduling, geographical location, professional curriculum requirements, and administrative buy-in. Practicing SBI skills in clinical sites with preceptors present is a unique experience that is highly valued by students.

## A49 Barriers and facilitators of implementation of screening, brief intervention and referral to treatment (SBIRT) for behavioral health problems in pediatric primary care: a qualitative interview study

### Stacy A. Sterling^1^, Ashley L. Jones^1^, Asheley C. Skinner^2^, Agatha Hinman^1^

#### ^1^Division of Research, Kaiser Permanente Northern California Division of Research, Oakland, CA, USA; ^2^Department of Medicine, Clinical Research Institute, Duke University, Durham, NC, USA

##### **Correspondence:** Stacy A. Sterling - stacy.a.sterling@kp.org

*Addiction Science & Clinical Practice* 2017, **12(Suppl 1)**: A49

**Background:** Pediatric primary care is an opportune setting for Screening, Brief Intervention and Referral to Treatment (SBIRT), but it has not been widely implemented, and there is little research on factors contributing to its implementation. This study used data from qualitative interviews (n = 20) with Kaiser Permanente Northern California (KPNC) and community-based pediatric primary care, specialty mental health and substance abuse treatment clinicians, policymakers and staff to examine factors which may inhibit or facilitate implementation of SBIRT in pediatric primary care. We used the Consolidated Framework for Implementation Research (CFIR) to inform our analysis, which used an inductive approach to build an understanding of the complexities involved in SBIRT implementation in pediatric primary care.

**Materials and methods:** Audiotapes of interviews were transcribed, and using NVivo software, were double-coded, independently, by coders blind to each other’s coding. Themes were created based on the broad constructs of the CFIR model: outer setting, inner setting, characteristics of the intervention, characteristics of the individuals involved, and process of implementation. Within those overarching constructs, SBIRT-specific sub-themes were developed, based on participant responses and informed by the extant literature. Percentage coder agreement and a Kappa Coefficient were calculated to measure inter-rater reliability, by interview, node, and across the sample.

**Results:** Inner setting factors, such as time, screening instruments and weak linkages between department and organizations were most frequently discussed as barriers to SBIRT implementation. Outer setting factors such as confidentiality laws and societal attitudes about substance use were also frequently cited as influencing implementation, as were patient characteristics, such as co-occurring mental health concerns and linguistic needs. Many respondents also discussed the role of clinician training and SBIRT skills. The single most frequently discussed implementation facilitator was adopting an integrated, embedded-behavioral health clinician model of SBIRT. Inter-rater reliability was high: Kappa coefficients ranged from 0.78 to 0.85, and percent agreement between coders was >98% across the constructs.

**Conclusions:** Participants identified many challenges to SBIRT implementation, but responses suggested a number of pragmatic policy and care delivery changes which could be taken to encourage its greater adoption.

## A50 Computer-delivered indirect screening and brief intervention for drug use in the perinatal period: a randomized trial

### Steven J. Ondersma^1^, Dace S. Svikis^2^, Casey L. Thacker^3^, Ken Resnicow^3^, Jessica R. Beatty^4^, James Janisse^5^, Karoline Puder^6^

#### ^1^Department of Psychiatry and Behavioral Neurosciences and Merrill-Palmer Skillman Institute, Wayne State University, Detroit, MI, USA; ^2^VCU Institute for Women’s Health and Departments of Psychology, Psychiatry, and Obstetrics/Gynecology, Virginia Commonwealth University, Richmond, VA, USA; ^3^School of Public Health, University of Michigan, Ann Arbor, MI, USA; ^4^Merrill-Palmer Skillman Institute, Wayne State University, Detroit, MI, USA; ^5^Department of Family Medicine and Public Health Sciences, Wayne State University, Detroit, MI, USA; ^6^Department of Obstetrics and Gynecology, Wayne State University, Detroit, MI, USA

##### **Correspondence:** Steven J. Ondersma - s.ondersma@wayne.edu

*Addiction Science & Clinical Practice* 2017, **12(Suppl 1)**: A50

**Background:** Under-reporting of drug use in the perinatal period is well-documented, and significantly limits brief intervention reach. The Wayne Indirect Drug Use Screener (WIDUS) is a validated screener focusing on correlates of drug use rather than drug use itself. The present trial tested the efficacy of a single-session computer-delivered screening and brief intervention designed for use with indirect screen-positive cases; this intervention sought to motivate reductions in substance use without presuming its presence. It did so by engaging participants in a tailored review of “parenting strengths” that are associated with positive child outcomes, one of which was “a healthy home” (defined as absence of substance abuse); other specified strengths, such as safety and emotional health, were selected in part because of their negative association with substance use. Participants were invited to consider whether they could enhance their or “their home’s” strengths in any of those areas. Participants who indicated interest in change were helped to set specific goals and identify resources for facilitating change (such as treatment).

**Materials and methods:** Randomized clinical trial with 500 WIDUS-positive postpartum women. Participants were randomly assigned to either a time control condition or a single-session, tailored brief intervention. The primary outcome was days of drug use over the 6-month follow-up period; secondary outcomes included urine and hair analysis results at 3- and 6-month follow-up.

**Results:** 36.1% of participants acknowledged drug use in the 3 months prior to pregnancy, but 89% tested positive for drugs at the 6-month follow-up. Participants rated the intervention as easy to use (4.9/5) and helpful (4.4/5). Analyses revealed no between-group differences in drug use. Exploratory analyses also showed that intervention effects were not moderated by baseline severity, WIDUS score, or readiness to change.

**Conclusions:** The present trial showed no evidence of efficacy for an indirect, single-session, computer-delivered brief intervention designed as a complement to indirect screening. More direct approaches that still do not presume active drug use may be possible and appropriate.

## A51 Innovative strategies for brief interventions targeting alcohol dependence: program development and implementation for treatment in primary care

### Sven Andréasson, Ann-Sofie Bakshi

#### Department of Public Health Sciences, Karolinska Institutet, Stockholm, Sweden, Stockholm, Sweden

##### **Correspondence:** Sven Andréasson - sven.andreasson@gmail.com

*Addiction Science & Clinical Practice* 2017, **12(Suppl 1)**: A51

**Background:** Alcohol use disorders are common, although a majority of affected individuals are reluctant to seek and undergo treatment in addiction care units. A major reason for this is the stigma attached to alcohol problems and treatment, generating a need to develop and implement stigma reducing interventions. Consequently, we developed the program’ treatment for alcohol dependence in primary care (TAP)’ for brief intervention tailored to the primary care setting. TAP was implemented in 12 primary care units in Stockholm, Sweden. The aim of the present study is to investigate the functionality of the TAP-program, as experienced by the participating practitioners.

**Materials and methods:** Data were collected through semi-structured interviews with general practitioners, working in primary care units participating in TAP. Ten informants were interviewed at baseline after they had attended a one-day training in TAP and before program implementation. Follow-up interviews were conducted after 6 months. The interviews were recorded, transcribed and analyzed through qualitative thematic content analysis.

**Results:** At baseline, informants expressed a need for increased knowledge on how to treat patients with alcohol disorders. They observed that several patients seemed to have problematic alcohol consumption but lacked a systematic method for interventions. In the follow-up study, the informants stated that TAP had provided a systematic treatment tool, which functioned well in the primary care setting. The program was perceived as potentially effective in reducing alcohol consumption and as resource effective. Furthermore it was considered uncomplicated and easy to use. Also, the informants were motivated to integrate it into the regular practice of their primary care units.

**Conclusion:** Knowledge gained from the TAP study can be used to further develop and implement innovative treatment strategies for alcohol problems. Importantly, the stigma problem, which to a large extent generates a threshold for seeking help, can be reduced. TAP is tailored to primary care and the results of the study indicate that primary care practitioners can be effectively engaged to deliver treatment for alcohol dependence.

## A52 Optimizing engagement with eSBI: The BRANCH app targeting harmful drinking in young adults

### Joanna M. Milward, Paolo Deluca, Andreas Kimergard, Colin Drummond

#### Addictions Department, King’s College London, London, UK

##### **Correspondence:** Joanna M. Milward - joanna.milward@kcl.ac.uk

*Addiction Science & Clinical Practice* 2017, **12(Suppl 1)**: A52

**Background:** Electronic screening and brief intervention (eSBI) smartphone apps demonstrate potential to reduce harmful drinking. However, low user engagement rates with eSBI reduce overall effectiveness. The Alcohol Theme of the Collaboration for Leadership in applied Health Research and Care (CLAHRC) South London, has developed an eSBI app targeting harmful drinking in young adults which included strategies for optimising engagement. The app, called ‘BRANCH’, was evaluated with a mixed-methods design, including a randomised controlled trial (RCT), which compared a basic and enhanced version of the app, as well qualitative interviews with participants from the RCT. The qualitative component, exploring participants’ engagement with the app is presented here.

**Materials and methods:** Qualitative 1:1 interviews were conducted with participants recruited from the basic and enhanced arms of the BRANCH RCT. Half of the participants were high engagers (logged in more than twice), the other half low engagers (logged in less than twice). Interviews explored participants’ experiences of using the app, including barriers and facilitators to engagement. A detailed thematic analysis was undertaken.

**Results:** Twenty participants were recruited from January to March 2017. Findings suggest that eSBI for young people is successful for specific user-types who are motivated to monitor their health and enjoy entering physical health data. Ease-of-access and low data-entry burden costs directly affected users’ engagement with the app.

**Conclusions:** This is the first study to explore participant engagement with an eSBI specifically targeting harmful drinking in young adults. ESBI may be appropriate for specific user groups of digital technology. Young people are proficient technology users who want simple and fast interactions with eSBI apps. If eSBI apps do not meet these expectations then young people quickly cease use altogether. Implications of the findings to improve engagement are discussed in the context of future app development.

## A53 Estimating effectiveness of components of a smartphone app- Drink Less—to reduce excessive alcohol consumption: a factorial randomised control trial

### Claire V. Garnett^1^, David Crane^1^, Jamie Brown^2^, Robert West^2^, Susan Michie^1^

#### ^1^Department of Clinical, Educational and Health Psychology, University College London, London, England; ^2^Department of Behavioural Science and Health, University College London, London, England

##### **Correspondence:** Claire V. Garnett - c.garnett.12@ucl.ac.uk

*Addiction Science & Clinical Practice* 2017, **12(Suppl 1)**: A53

**Background:** Smartphone apps have the potential to help drinkers reduce hazardous and harmful alcohol consumption. However, there have been few evaluations of the effectiveness of these apps and none to our knowledge that estimates the effects of individual intervention components. This study aimed to evaluate the effectiveness of intervention components of an alcohol reduction app, Drink Less.

**Materials and methods:** Drink Less is a freely available app to any individual in the UK making an attempt to reduce their drinking. The app was structured around goal setting with information on the UK drinking guidelines, units and alcohol-related harms. The app offered access to five additional intervention modules—Normative Feedback, Cognitive Bias Re-training, Self-monitoring and Feedback, Action Planning and Identity Change—to help them achieve their goal. Excessive drinkers (AUDIT ≥ 8) who were aged 18+ were orthogonally randomised to receive ‘enhanced’ or ‘minimal’ versions of each of the five modules (to a total of 25 experimental conditions). The primary outcome measure was change in past week consumption at one-month follow-up. Secondary measures were change in AUDIT score, usage data and usability ratings. A factorial between-subjects ANOVA assessed main and interactive effects of the app modules using an intention-to-treat analysis.

**Results:** Of 672 study participants, 27% responded to follow-up. At baseline, the mean past week consumption was 39.9 units (SD = 27.34) and mean AUDIT score was 19.1 (SD = 6.56). There were no significant main effects of the intervention modules on either measure. There were two-way interactions between enhanced Self-monitoring and Feedback and Action Planning on AUDIT score (F = 5.818, *p* = 0.016) and between enhanced Normative Feedback and Cognitive Bias Re-training on past week consumption (F = 4.676, *p* = 0.031). Enhanced Self-monitoring and Feedback was used more often and rated more positively for helpfulness, satisfaction and recommendation than the minimal version.

**Conclusions:** Individual enhanced modules were not more effective compared with their minimal condition. The combinations of Self-monitoring and Feedback with Action Planning, and Normative Feedback with Cognitive Bias Re-training resulted in significant reductions in alcohol-related outcomes when both modules were enhanced. Users rated the Self-monitoring and Feedback module significantly more positively when it was enhanced.

## A54 Smartphone apps targeting risky and excessive drinking patterns among university students show differing subgroup effects over 20 weeks

### Anne H. Berman^1,2^, Ingvar Rosendahl^1^, Claes Andersson^3^, Mikael Gajecki^1,2^, Kristina Sinadinovic^1^, Matthijs Blankers^4,5,6^

#### ^1^Department of Clinical Neuroscience, Center for Psychiatry Research, Karolinska Institutet, Stockholm, Sweden; ^2^Stockholm Center for Dependency Disorders, Stockholm, Sweden; ^3^Malmö University, Department of Criminology, Malmö, Sweden; ^4^Trimbos Institute—The Netherlands Institute of Mental Health and Addiction, Utrecht, The Netherlands; ^5^Arkin Mental Health Care, Amsterdam, The Netherlands; ^6^Department of Psychiatry, Academic Medical Centre, University of Amsterdam, Amsterdam, The Netherlands

##### **Correspondence:** Anne H. Berman - anne.h.berman@ki.se

*Addiction Science & Clinical Practice* 2017, **12(Suppl 1)**: A54

**Background and Aims:** University students with risky drinking are a clear target group for intervention via smartphone apps. This study compared three different apps over a 20-week period, for university students with hazardous and excessive drinking patterns.

**Materials and Methods:** Students from six campuses were invited to a three-armed trial (A). Those with hazardous alcohol use (n = 2166) were randomly assigned to one of two smartphone apps offering feedback on real-time estimated blood alcohol concentration (eBAC) levels, or to a control group, with three follow-ups at 6, 12 and 20 weeks. At 6 weeks, participants in the app groups with excessive weekly alcohol consumption of >9 (women) or >14 (men) drinks per week (n = 257), were offered participation in a second trial (B); consenters (n = 186) were randomly assigned to a skills-based app or a waitlist group, and compared with an assessment-only control group.

**Results:** Six-week analyses (n = 2166) replicated our earlier trial from 2014, re-confirming earlier results: the Promillekoll app was associated with higher quantity and frequency of drinking compared to controls, and a higher risk for excessive drinking; the PartyPlanner group did not differ from controls. Lower-risk drinkers from trial A (n = 1177) up to 20 weeks did not differ from controls on main outcomes. However, sub-analyses showed that individuals with higher consumption had higher motivation to reduce intake. In both intervention groups, consumption was lower for more highly motivated participants compared to controls at 6- and 20-week follow-ups. Latent class analysis of participants in both trials (n = 2166) revealed a class (n = 146) that drank several days a week and that differed significantly from the remaining cohort in gender, age, and alcohol consumption. For this class, access to the Promillekoll app appeared marginally associated with lower quantity over time; access to the skills-based TeleCoach app was clearly associated with fewer drinking days up to 20 weeks.

**Conclusions:** Smartphone apps targeting eBAC can influence drinking levels up to 20 weeks for university students with hazardous use and higher motivation to reduce their drinking. A skills-based app that reduces intake among students with excessive weekly consumption can be particularly effective for students with daily drinking habits.

## A55 Effectiveness and cost effectiveness of a smartphone based electronic alcohol intervention for adolescents: findings from the SIPS jr trials

### Paolo Deluca^1^, Simon Coulton^2^, Sadie Boniface^1^, Kim Donoghue^1^, Eilish Gilvarry^3^, Eileen Kaner^4^, Ellen Lynch^4^, Ian Maconochie^5^, Ruth McGovern^4^, Dorothy Newbury-Birch^6^, Ceri Phillips^7^, Rhys Pockett^7^, Tom Phillips^1^, R. Patton^8^, Ian Russell^9^, John Strang^1^, Colin Drummond^1^

#### ^1^Department of Addictions, Institute of Psychiatry, Psychology and Neuroscience, King’s College London, London, UK; ^2^Centre for Health Services Studies, University of Kent, Canterbury, Kent, UK; ^3^Northumberland, Tyne and Wear NHS Foundation Trust, UK; ^4^Institute of Health and Society, Newcastle University, Newcastle, UK; ^5^Paediatric Emergency Medicine, Imperial College London, London, UK; ^6^School of Health and Social Care, Teesside University, Middlesbrough, UK; ^7^Swansea Centre for Health Economics, College of Human and Health Sciences, Swansea University, Swansea, Wales, UK; ^8^School of Psychology, University of Surrey, Guildford, UK; ^9^Swansea University Medical School, Swansea, Wales, UK

##### **Correspondence:** Paolo Deluca - Paolo.Deluca@kcl.ac.uk

*Addiction Science & Clinical Practice* 2017, **12(Suppl 1)**: A55

**Background:** This paper reports two linked randomized controlled trials which aimed to evaluate the effectiveness and cost-effectiveness of two BI intervention strategies among adolescents attending Emergency Departments (EDs), compared with screening alone. One trial focused on high-risk adolescent drinkers and the other focused on those identified as low-risk or abstinent from alcohol. In both trials our primary outcome measure was quantity of alcohol consumed at 12 months after randomization. Our primary (null) hypothesis was: Personalized Feedback and Brief Advice (PFBA) and Personalized Feedback plus electronic Brief Intervention (eBI) are no more effective than screening alone in reducing alcohol consumed at 12 months after randomization measured by the AUDIT-C.

**Materials and methods:** 1640 adolescents (aged 14–17) attending ten EDs across three regions of England were screened for alcohol consumption and randomized into the trials. 73% across the two trials were follow up at 12 months.

**Results:** In both trials no significant differences in outcome were found between groups on either primary or secondary outcome measures. This supported the null hypothesis that PFBA and eBI are no more effective nor cost effective in reducing alcohol consumption in low-risk drinkers than screening alone. For those allocated to eBI, 34% actually engaged with the intervention after leaving the ED. No relationship was identified between engagement with the intervention and alcohol consumption at month 12. However, females were more likely to download the app than males (*p* < 0.001).

**Conclusions:** In both trials we found that engagement with the eBI intervention was low in participants randomized to eBI. Only a third of participants engaged with the eBI platform after leaving ED. This may have limited the impact of the eBI intervention compared to control intervention. However, as these were pragmatic trials, this is likely to be the level of engagement expected in the typical patient recruited for ED.

## A56 Reducing risky drinking: what health care systems can do

### Maureen T. Stewart^1^, Amity E. Quinn^2^, Mary Brolin^1^, Brooke Evans^1^, Constance Horgan^1^

#### ^1^Institute for Behavioral Health, The Heller School for Social Policy and Management, Brandeis University, Waltham, MA, USA; ^2^Department of Community Health Sciences, Cumming School of Medicine, University of Calgary, Calgary, AB, Canada

##### **Correspondence:** Maureen T. Stewart - mstewart@brandeis.edu

*Addiction Science & Clinical Practice* 2017, **12(Suppl 1)**: A56

**Background:** Risky, non-dependent alcohol use is prevalent in the United States, and Massachusetts has higher rates of alcohol use and binge drinking than other states. Screening and brief intervention (SBI) is an evidence-based practice to address risky alcohol use, but numerous barriers are slowing the rate of SBI uptake. While healthcare providers play a critical role in increasing the use of alcohol SBI, health care systems have a role encouraging further expansion. The goal of this project was to bring health care system stakeholders together to identify actionable steps to advance alcohol SBI and improve population health in Massachusetts.

**Materials and methods:** First, results from a national survey of health plan activities related to SBI were synthesized with literature review and analysis. Next, a public forum was convened and a policy brief was presented to provide education and motivation regarding the importance of SBI. Finally, Massachusetts health care stakeholders, representing health plans, provider groups, state and federal government, and researchers came together to discuss how to advance alcohol SBI efforts in the state and qualitative analyses were conducted.

**Results:** A number of key issues and next steps were identified, which fell into strategies for providers and those for health plans. For providers, the importance of utilizing non-physician staff for SBI delivery, improving medical education, and offering a toolbox for primary care providers to use were noted. Strategies for health plans included the plans themselves conducting screening, improving performance measurement—plans were particularly interested in a possible HEDIS alcohol screening measure, payment reform, and support for referral to treatment. Finally, strategies for other stakeholders to advance SBI included partnerships with state and federal organizations, researcher support and assistance, public education campaigns, and working with consumer groups to harness community support.

**Conclusions:** Providers, delivery systems, and payers all have roles to play in improving how risky drinking is addressed in the state. Next steps identified by stakeholders included: scheduling follow-up meetings, developing and disseminating SBI and referral implementation and practice guidelines, offering training and support for providers and health care systems in the state, and improving performance measurement.

## A57 Low screening and follow-up for unhealthy alcohol use in Medicaid health plans

### Junqing Liu^1^, Fern McCree^1^, Doug Kanovsky^1^, Tyler Oberlander^1^, Huan Zhang^1^, Ben Hamlin^1^, Robert Saunders^1^, Mary B. Barton^1^, Sarah H. Scholle^1^, Patricia Santora^2^, Chirag Bhatt^3^, Kazi Ahmed^3^

#### ^1^National Committee for Quality Assurance, Washington, DC, USA; ^2^The Substance Abuse and Mental Health Services Administration, Rockville, MD, USA; ^3^FEi systems, Columbia, MD, USA

##### **Correspondence:** Junqing Liu - liu@ncqa.org

*Addiction Science & Clinical Practice* 2017, **12(Suppl 1)**: A57

**Background:** National studies report that about 20% of adults engage in hazardous drinking. The US Preventive Services Task Force (USPSTF) recommends that providers screen adults for alcohol misuse. Many state Medicaid programs cover screening, but little is known about Medicaid plan performance. This pilot study examines the rate of screening and follow-up for unhealthy alcohol use among Medicaid enrollees.

**Materials and methods:** We used 2015 data submitted by two Medicaid plans. We adapted the American Medical Association’s measure, Unhealthy Alcohol Use: Screening and Brief Counseling, for health plan reporting. Additional testing of this measure was undertaken to determine whether it met requirements for inclusion in the Healthcare Effectiveness Data and Information Set (HEDIS). We examined the percentage of adult members who received unhealthy alcohol use screening using a recommended standardized tool (e.g., AUDIT, AUDIT-C) and received follow-up care if they screened positive. We studied annual screening and investigated follow-up care within three months after a positive screening, using a random sample of 108 (Plan A) and 120 (Plan B) adult members. Plans provided de-identified, member-level data based on case management records, claims and manual chart review of electronic health records (EHR).

**Results:** 40% of Plan A members were screened in 2015 for alcohol use; 29% screened positive. 33% of members who screened positive received follow-up care, generally within two months after screening. 38% of Plan B members were screened in 2015; one member had documented positive results and received follow-up care within two months. Screening rates were based on non-standardized screening tools because standardized tools were rarely used. Screening and follow-up care were rarely documented in structured EHR fields. Claims or case management data alone would find only half the care provided.

**Conclusions:** Despite national recommendations, rates of screening and follow-up for unhealthy alcohol use are low in Medicaid populations. USPSTF-recommended standardized tools are rarely used; results are seldom documented in structured electronic data. Structured EHR fields for standardized screening tools and follow-up care will allow easier monitoring of care quality. Provider education, greater access to treatment and quality measure reporting can encourage better care for alcohol misuse.

## A58 Implementing single-item screening for drug use in a Veterans Administration outpatient setting

### Dominic Hodgkin^1^, Wenwu Gao^2^, Elizabeth L. Merrick^1^, Charles E. Drebing^3^, Mary Jo Larson^1^, Constance M. Horgan^1^, Monica Sharma^4^, Nancy M. Petry^5^, Richard Saitz^6^

#### ^1^Institute for Behavioral Health, Schneider Institutes for Health Policy, Heller School for Social Policy and Management, Brandeis University, Waltham, MA, USA; ^2^Psychology Service, Bedford Department of Veterans Affairs Medical Center, Bedford, MA, USA; ^3^Psychology Service, Bedford Department of Veterans Affairs Medical Center, Bedford, MA, USA; ^4^Primary Care Service, Bedford Department of Veterans Affairs Medical Center, Bedford, MA, USA; ^5^University of Connecticut School of Medicine, CT, USA; ^6^Department of Community Health Sciences, Boston University School of Public Health, Boston, MA, USA

##### **Correspondence:** Dominic Hodgkin - hodgkin@brandeis.edu

*Addiction Science & Clinical Practice* 2017, **12(Suppl 1)**: A58

**Background:** Unhealthy drug use is a concern in many settings, including military and veteran populations. In 2013, the Veterans Administration (VA) medical center in Bedford, Massachusetts, started requiring routine annual screening for unhealthy drug use in outpatient primary care and mental health settings, using a validated single question. This new policy builds on prior experience with annual screening for unhealthy alcohol use, which the VA has required of physicians since 2004.

**Materials and methods:** This study used descriptive and multivariable analyses of VA administrative data for patients eligible for screening (N = 16,118). The study assessed first-year rates and predictors of screening and of positive screens, both for drug use and for unhealthy alcohol use, for which screening was already required.

**Results:** During the first year, 70% of patients were screened for drug use, and 84% were screened for unhealthy alcohol use. In multivariable analyses, screening for drug use was more likely for patients who had 8 or more days with VA visits or were aged 60 or over. Patients with a prior drug use disorder diagnosis were much less likely to be screened. Strong predictors of a positive drug use screen included a prior diagnosis of drug use disorder, prior mental health visits, younger age or being unmarried.

**Conclusions:** The drug screening initiative was relatively successful in its first-year implementation, having screened 70% of eligible subjects. This proportion was somewhat lower than the 84% achieved for the longer-established alcohol screening program. Importantly, physicians failed to screen for drug use many of those patients who were most likely to screen positive, thereby missing many opportunities to treat and refer patients with drug use disorders. Future refinements should include better training clinicians in how to ask sensitive questions and how to deal with positive screens.

## A59 Universal versus targeted screening for tobacco and drug use in adult primary care

### Constance M. Weisner, Kelly C. Young-Wolff, Wendy Y. Lu, Felicia W. Chi, Stacy A. Sterling

#### Division of Research, Kaiser Permanente, Oakland, CA, USA

##### **Correspondence:** Constance M. Weisner - Constance.Weisner@kp.org

*Addiction Science & Clinical Practice* 2017, **12(Suppl 1)**: A59

**Background:** The optimal strategy for accomplishing screening and intervention for all substances in primary care has not been found. One question has to do with the comprehensiveness of using hazardous drinking as the foundation, with further screening for other substances of those who meet hazardous drinking criteria. Aims: To explore the relative efficiency of targeted versus universal screening of tobacco use disorders, drug use disorders and opioid-at-risk use in adult primary care.

**Materials and methods:** Kaiser Permanente Northern California has incorporated alcohol SBIRT into adult primary care. A total of; 2312,922 adults were screened for unhealthy drinking during 2014–2015. Of those, 253,821 (11%) screened positive (i.e., exceeding daily or weekly limits). We compared rates of diagnosis-based tobacco use and drug use disorders, and opiate-at-risk use (prescription patterns) among all patients screened for unhealthy drinking (universal) versus those screened positive (targeted).

**Results:** The proportion of tobacco use disorders was 7.0% among all adults who were screened and 12.1% among those exceeding either daily or weekly limits. The prevalence ranged across age groups (12.7–20.6%) and genders (females 17.7%, males 19.9%), and were much higher for those exceeding both daily and weekly limits. On the other hand, only 4% of those with tobacco disorders reported exceeding both daily and weekly drinking limits.

Proportions of drug use disorders were similar using both approaches: 1.8% among all patients screened, and 2.3% among those exceeding daily or weekly limits. Proportions were higher among those exceeding weekly limits, especially those aged 18–25 and 26–44 for whom the proportions were twice as high as from universal screening. However, the vast majority of those with drug disorders (86%) would have been missed by the targeted screening approach. The proportion of those meeting opioid-at-risk use criteria was lower among those who screened positive for hazardous drinking (3.8%) than among all patients screened (5.4%); findings were similar across demographic groups. We will conduct multivariate analyses focusing on each substance, examining patient characteristics.

**Conclusion:** A limitation is that we measured “disorders” rather than “at-risk” use, except for opioids. Aside from tobacco, targeted screening was not comprehensive; strategies are needed for more complete screening.

## A60 Receipt of alcohol screening and brief intervention among transgender and non-transgender adults in the U.S.: exploratory findings from a national health survey

### John R. Blosnich^1^, Keren Lehavot^2^, Joseph E. Glass^3^, Emily C. Williams^2^

#### ^1^Department of Veterans Affairs, VA Pittsburgh Healthcare System, Center for Health Equity Research and Promotion, Pittsburgh, PA, USA; ^2^Denver-Seattle Center of Innovation for Veteran-Centered Value-Driven Care, VA Puget Sound Healthcare System, Seattle, WA, USA; ^3^Kaiser Permanente Washington Health Research Institute, Seattle, WA, USA

##### **Correspondence:** John R. Blosnich - john.blosnich@va.gov

*Addiction Science & Clinical Practice* 2017, **12(Suppl 1)**: A60

**Background:** Prior research shows that many transgender individuals (persons whose current gender identity is different from their sex assigned at birth) must teach their healthcare providers about transgender healthcare delivery, and transgender individuals experience discrimination and harassment within the healthcare system. Consequently, it is unclear whether transgender individuals are less likely to receive alcohol screening and brief intervention during clinical visits in comparison with their non-transgender peers.

**Materials and methods:** Data are from the 2014 Behavioral Risk Factor Surveillance System surveys of eight US states that administered both the Alcohol Screening and Brief Intervention (ASBI) module and the Sexual Orientation and Gender Identity module. The analytic sample (n = 49,208) included all individuals who provided their gender identity and had a medical checkup in the last two years (a prerequisite for the ASBI module). Analyses were conducted in 2016–2017.

**Results:** A weighted proportion of 0.6% (n = 283) of the sample self-identified as transgender. No significant differences in alcohol consumption (i.e., any alcohol use in the past 30 days, heavy alcohol use, heavy episodic drinking) were observed between transgender and non-transgender groups. Transgender and non-transgender groups did not differ in being asked by a healthcare provider if they drink alcohol, how much alcohol they drank, or if they had any heavy episodic drinking. Although a greater proportion of transgender than non-transgender individuals (31.7 vs. 19.9%) reported being offered advice about unhealthy alcohol use, this difference was not statistically significant (*p* = .054). Compared with non-transgender individuals, transgender individuals who received alcohol use screening were more likely to report being advised to quit or reduce their drinking (20.7 vs. 7.8%; *p* = .012); but adjustment for socio-demographics attenuated this difference (adjusted odds ratio = 2.31, 95% CI 0.91–5.86, *p* = .077).

**Conclusions:** This is the first study on provision of alcohol-related care for transgender individuals among a population-based sample with a direct non-transgender comparison group. Results did not identify significant differences between groups, and in the rare instances differences were observed, findings suggested transgender individuals received more brief interventions than their non-transgender peers. Differences notwithstanding, alcohol screening and brief intervention should be increased among both groups.

## A61 Alcohol use and receipt of alcohol screening and brief intervention in a representative sample of sexual minority and heterosexual adults receiving health care

### Keren Lehavot^1^, John R. Blosnich^2^, Joseph E. Glass^3^, Emily C. Williams^1^

#### ^1^Denver-Seattle Center of Innovation for Veteran-Centered Value-Driven Care, VA Puget Sound Healthcare System, Seattle, WA, USA; ^2^Department of Veterans Affairs, VA Pittsburgh Healthcare System, Center for Health Equity Research and Promotion, Pittsburgh, PA, USA; ^3^Kaiser Permanente Washington Health Research Institute, Seattle, WA, USA

##### **Correspondence:** Keren Lehavot - keren.lehavot@va.gov

*Addiction Science & Clinical Practice* 2017, **12(Suppl 1)**: A61

**Background:** Sexual minorities, including lesbian, gay, and bisexual women and men, experience a wide array of health disparities and have recently been designated as a health disparity population by the National Institutes of Health. Despite evidence of alcohol disparities between sexual minority and heterosexual individuals in the general population, research has not examined whether there are disparities in receipt of alcohol screening and brief intervention—together considered one of the highest prevention priorities for US adults. This study examined differences in alcohol use and receipt of alcohol screening and brief intervention across sexual minority status.

**Materials and methods:** Behavioral Risk Factor Surveillance System 2014 data from eight US states were used to estimate patterns of alcohol use and receipt of alcohol screening and brief intervention among persons reporting sexual orientation and a checkup in the last two years (N = 47,800). Analyses were conducted in 2016–2017.

**Results:** Gay men had a higher age-adjusted prevalence of any alcohol use compared to heterosexual men in the past 30 days (79.5 vs. 60.1%), and bisexual women trended toward significance in having a higher prevalence of risky drinking (14.4 vs. 4.9%), heavy episodic drinking (21.1 vs. 10.8%), and any unhealthy alcohol use (22.8 vs. 12.0%) compared to heterosexual women in the past 30 days. We found few differences in receipt of alcohol screening and brief intervention by sexual orientation. Specifically, lesbian women were more likely to report being asked about heavy episodic drinking than heterosexual women during a checkup, and among those reporting unhealthy alcohol use, gay men were less likely, and bisexual men were more likely, to report receiving brief intervention compared to heterosexual men.

**Conclusions:** Overall similarities between sexual minorities and heterosexuals in alcohol use and receipt of screening and brief intervention are encouraging. Nonetheless, a potential area of disparity emerged with respect to gay men’s lower likelihood of being offered advice about drinking compared to heterosexual men among those for which brief interventions are indicated. Future research is needed to understand mechanisms underlying this disparity, as well as to assess variation in quality of alcohol-related care across sexual minority status.

## A62 Racial/ethnic and gender differences in receipt of brief intervention among patients with unhealthy alcohol use in the U.S. Veterans Health Administration

### Emily C. Williams^1^, Joseph E. Glass^2^

#### ^1^Department of Health Services, University of Washington School of Public Health, Seattle, WA, USA; ^2^Kaiser Permanente Washington Research Institute, Seattle, WA, USA

##### **Correspondence:** Emily C. Williams - Emily.Williams3@va.gov

*Addiction Science & Clinical Practice* 2017, **12(Suppl 1)**: A62

**Background:** Intersectionality theory posits that individuals with multiple marginalized social statuses are likely to be at magnified risk for poor health and health care than those with one or no marginalized social statuses. While previous studies have reported gender and racial/ethnic differences in receipt of brief intervention for unhealthy alcohol use, no study has assessed the intersection of race and gender in association with receipt of brief intervention. In a national sample of patients with unhealthy alcohol use, we examined receipt of brief intervention across race/ethnicity and gender.

**Materials and methods:** VA outpatients who had one or more positive screens for unhealthy alcohol use (AUDIT-C ≥ 5) documented in their medical records between 10/2009 and 5/2013 were eligible. Poisson regression models with a race by gender interaction were fit to identify the predicted prevalence and 95% confidence intervals (CI) of having brief intervention documented ≤14 days after a positive alcohol screen for American Indian, Asian/Pacific Islander, black, Hispanic, and white men and women. Models included a robust sandwich estimator to account for correlation resulting from multiple screens.

**Results:** The sample included 830,825 outpatients (3% women, 25% non-white), reflecting 1,172,606 positive screens (1–5 per patient); 74% had documented brief intervention. Both American Indian and black patients were less likely than white patients to receive brief intervention (*p*-values <0.001), as were women relative to men (*p* < 0.001), and a significant interaction between race and gender was identified (*p* < 0.001). The predicted prevalence of receiving brief intervention ranged from 66.8% (95% CI 65.7–67.8) among black women to 74.7% among Asian/Pacific Islander (95% CI 74.6–74.8), Hispanic (95% CI 74.0–75.1), and white (95% CI 74.6–74.8) men.

**Conclusions:** Race and gender, individually and intersectionally, appear to influence receipt of brief intervention among patients with unhealthy alcohol use. Black women with unhealthy alcohol use appear to be at the greatest risk of under-receipt of brief intervention. Future research should investigate mechanisms underlying these associations.

## A63 Differences in receipt of alcohol-related care across race/ethnicity among VA patients living with HIV and unhealthy alcohol use

### Kara M. Bensley^1^, India Ornelas^1^, Gary Chan^2^, Julie Dombrowski^3^, John Fortney^4^, Anna D. Rubinsky^5^, Gwen T. Lapham^6^, Joseph E. Glass^6^, Emily C. Williams^1^

#### ^1^Department of Health Services, University of Washington, Seattle, WA, USA; ^2^Department of Health Services and Department of Biostatistics, University of Washington, Seattle, WA, USA; ^3^Department of Medicine and Allergy and Infectious Diseases, University of Washington, WA, USA; ^4^Department of Psychiatry and Behavioral Sciences, University of Washington, and Health Services Research and Development, Veterans Health Administration Puget Sound, Seattle, WA, USA; ^5^Kidney Health and Research Collaborative, University of California San Francisco, San Francisco, CA, USA; ^6^Kaiser Permanente Washington Health Research Institute, Seattle, WA, USA

##### **Correspondence:** Kara M. Bensley - kbensley@uw.edu

*Addiction Science & Clinical Practice* 2017, **12(Suppl 1)**: A63

**Background:** Unhealthy alcohol use (UAU) is particularly risky for patients living with HIV (PLWH) and associated with worse outcomes for racial/ethnic minorities. While evidence-based interventions for UAU are available, whether receipt of such interventions is equitable across racial/ethnic groups among PLWH is unknown.

**Materials and methods:** VA electronic health record (EHR) data were used to identify alcohol screens positive for UAU (AUDIT-C ≥ 5) between 10/1/09 and 5/30/13 among black, Hispanic, and white patients with a past-year diagnosis for HIV and a documented home zip code. We measured three domains of evidence-based care from the EHR: brief intervention (advice to reduce or abstain from drinking) within 14 days of the screen among all positive screens; and specialty addictions treatment and pharmacotherapy (fills for naltrexone, disulfiram, acamprosate or topiramate) within one year of screening among those with diagnosed alcohol use disorder (AUD). Poisson regression models and recycled predictions were used to estimate adjusted predicted prevalence of receiving alcohol-related care. Models were adjusted for age, sex, rurality, VA eligibility status, marital status, and region and accounted for correlation within patients using a robust sandwich estimator.

**Results:** Among 3310 PLWH with UAU (4376 screens), 1968 were black, 257 were Hispanic, and 1085 were white; 2233 (50%) had AUD. Among all with UAU, no racial/ethnic differences in brief intervention were observed. Adjusted prevalences were 55.9% (95% CI 54.7–64.9%) among black, 59.8% (54.7–64.6%) among Hispanic, and 59.2% (56.4–61.9%) among white patients (*p* = 0.1223). Among those with AUD, adjusted prevalence of specialty addictions treatment differed across race/ethnicity with 52.1% (49.2%, 55.0%) for black patients, 39.2% (31.5%, 46.8%) for Hispanic patients, and 33.5% (29.3%, 37.7%) for white patients (*p* < 0.001). No differences in pharmacotherapy were observed; adjusted prevalences were 6.0% (4.6%, 7.4%) among black; 7.1% (2.2–11.9%) among Hispanic, and 6.5% (4.4–8.5%) among white patients (*p* = 0.7857).

**Conclusion:** Among patients with AUD, receipt of specialty addictions treatment appeared to be most common among black patients. No other racial/ethnic differences in alcohol-related care were identified. Identified differences are consistent with research in non-HIV specific samples but mechanisms underlying them are unknown and should be further investigated.

## A64 Electronic- and clinician-delivered screening, brief intervention, and referral to treatment for women in reproductive healthcare centers: a randomized clinical trial

### Kimberly A. Yonkers^1^, Steven J. Ondersma^2^, Ariadna Forray^1^, Todd A. Olmstead^3^, Kathryn Gilstad-Hayden^1^, Trace Kershaw^4^, Steve Martino^4^

#### ^1^Department of Psychiatry, Yale School of Medicine, New Haven, CT, USA; ^2^Department of Psychiatry and Behavioral Neurosciences and Merrill-Palmer Skillman Institute, Detroit, MI, USA; ^3^Lyndon B. Johnson School of Public Affairs, University of Texas at Austin, Austin, TX, USA; ^4^School of Epidemiology and Public Health, Yale School of Medicine, New Haven, CT, USA; ^4^Department of Psychiatry, Yale School of Medicine, VA Connecticut Healthcare, New Haven, CT, USA

##### **Correspondence:** Kimberly A. Yonkers - Kimberly.Yonkers@Yale.edu

*Addiction Science & Clinical Practice* 2017, **12(Suppl 1)**: A64

**Background:** Women are at the highest risk of misusing substances during their reproductive years. Given the fact that many women in this age group often use their reproductive health provider as their primary source of medical care, this setting may be an optimal place to screen and deliver a brief intervention to women who misuse substances. The goal of this project was to determine whether Screening, Brief Intervention, and Referral to Treatment delivered electronically (e-SBIRT) or by clinician (SBIRT) reduces substance misuse more than enhanced usual care (EUC) in women seeking routine care in a reproductive health setting.

**Materials and Methods:** Women (N = 439) from two reproductive healthcare clinics who smoked cigarettes or misused alcohol, illicit drugs, or prescription medication were randomized to a 20-min e-SBIRT, 20-min SBIRT, or EUC (receipt of a pamphlet with information and referrals). Assessments occurred at baseline and one, three, and six months post-baseline. Co-primary outcomes were days/month of primary substance use and post-intervention service utilization for participant reported primary substance.

**Results:** The number of participants randomized included: 143 to e-SBIRT, 145 to SBIRT, and 151 to EUC, with retention >84% at all points. At baseline, the mean (SD) days per month of primary substance use were 23.7 (7.7) for e-SBIRT, 23.2 (8.3) for SBIRT and 24.2 (7.7) for EUC, which respectively declined to 19.7 (11.2), 19.3 (11.2), and 22.8 (8.9) at one month, 17.8 (11.9), 18.0 (12.0), and 21.4 (10.6) at three months, and 16.2 (12.5), 17.0 (12.3), and 19.1 (11.8) at six months. Estimated declines were greater in e-SBIRT [β (SE) = −0.090 (0.034), *p* = 0.008; Cohen’s d = 0.19 at one month, 0.30 at three months, and 0.17 at six months] and SBIRT [β (SE) = −0.078 (0.037), *p* = 0.038; Cohen’s d = 0.17 at one month, 0.22 at three months, and 0.06 at six months] compared to EUC. Service utilization did not differ between groups.

**Conclusions:** E-SBIRT and SBIRT significantly decreased days of primary substance use among women in reproductive healthcare centers; neither SBIRT resulted in more service utilization than EUC.

## A65 Electronic and clinician-delivered screening, brief intervention, and referral to treatment for women in reproductive healthcare centers: economic analyses

### Steve Martino^1^, Todd A. Olmstead^2^, Steven J. Ondersma^3^, Ariadna Forray^4^, Kathryn Gilstad-Hayden^4^, Trace Kershaw^5^, Kimberly A. Yonkers^4^

#### ^1^Department of Psychiatry, Yale School of Medicine, VA Connecticut Healthcare System, West Haven, CT, USA; ^2^Lyndon B. Johnson School of Public Affairs, University of Texas at Austin, Austin, TX, USA; ^3^Department of Psychiatry and Behavioral Neurosciences and Merrill-Palmer Skillman Institute, Detroit, MI, USA; ^4^Department of Psychiatry, Yale School of Medicine, New Haven, CT, USA; ^5^School of Epidemiology and Public Health, Yale School of Medicine, New Haven, CT, USA

##### **Correspondence:** Steve Martino - steve.martino@yale.edu

*Addiction Science & Clinical Practice* 2017, **12(Suppl 1)**: A65

**Background:** The American College of Obstetricians and Gynecologists recommends the use of Screening, Brief Intervention, and Referral to Treatment (SBIRT) in reproductive healthcare centers to help women reduce or stop substance misuse. A recent trial showed both electronically-delivered SBIRT (e-SBIRT) and clinician-delivered SBIRT, when compared to enhanced usual care (EUC), resulted in a significant reduction in the days of primary substance use over a 6-month follow-up period. However, decision makers need guidance about the relative cost-effectiveness of these two approaches. This study presents the cost and cost-effectiveness of e-SBIRT and SBIRT relative to EUC in reducing substance misuse among women seeking routine care in reproductive health settings.

**Materials and methods:** Cost data were collected prospectively during the trial. Both variable and fixed costs for each condition were estimated from the provider and patient perspectives. Effectiveness was assessed in terms of the number of days per month of primary substance use during the 6-month follow-up period. Incremental cost-effectiveness ratios and cost-effectiveness acceptability curves were used to determine the relative cost-effectiveness of the three conditions.

**Results:** From the provider perspective, e-SBIRT dominated both EUC and SBIRT (i.e., on average, e-SBIRT both costs less and leads to more days of abstinence during the 6-month follow-up than EUC and SBIRT). From the societal perspective, (a) e-SBIRT dominates SBIRT, and (b) compared to EUC, the cost of using e-SBIRT to attain an additional day of abstinence is 14 cents.

**Conclusions:** e-SBIRT is a promising cost-effective approach for use in reproductive healthcare centers to help women reduce or stop substance misuse.

## A66 A RCT comparing computer and therapist-delivered SBIRT for alcohol/drugs in an urban primary care setting

### Dace S. Svikis^1^, Steven J. Ondersma^2^, Pamela Dillon^3^, Michael F. Weaver^4^

#### ^1^Department of Psychology; Institute for Women’s Health, Virginia Commonwealth University, Richmond, VA, USA; ^2^Merrill-Palmer Skillman Institute and Department of Psychiatry and Behavioral Neurosciences, Wayne State University, Detroit, MI, USA; ^3^Center for Clinical and Translational Research, Virginia Commonwealth University, Richmond, VA, USA; ^4^University of Texas Health Science Center, Houston, TX, USA

##### **Correspondence:** Dace S. Svikis - dssvikis@vcu.edu

*Addiction Science & Clinical Practice* 2017, **12(Suppl 1)**: A66

**Background:** The present study examined 3 and 6 month outcome data from a 4-arm randomized controlled trial comparing computer-directed and therapist-delivered brief interventions for heavy/problem drinking and drug use in patients attending an urban primary care clinic.

**Materials and methods:** Primary care patients with heavy/problem alcohol or drug use were identified using an anonymous computer health survey. Those providing informed consent (N = 713) were randomized to one of 4 study groups: computer-directed intervention (CACI), therapist-delivered intervention (CATI), assessment only control (CA), and minimal screen-only control (SC). SC participants answered no substance-use related questions until 3 and 6 month follow-up, when Timeline Follow-back (TLFB) data on alcohol and other drug use were obtained. The sample was 61% female and 77% African American, with mean age of 45.3 years. Analyses compared 7-day point prevalence rates of alcohol use, binge drinking and illicit drug use across the 4 study groups, stratified by the primary substance identified using a computer algorithm at baseline.

**Results:** The algorithm placed N = 343 (48%) of participants into a heavy alcohol use subgroup and N = 370 (52%) were placed in an illicit drug use subgroup. Within the alcohol use subgroup, 7-day point prevalence abstinence trends for any use favored the CACI condition (e.g., 55% abstinence at 6 months vs. 44, 41 and 42% for CATI, CA, and SC, respectively), but did not reach significance. Similarly, 7-day point prevalence binge abstinence also showed non-significant advantages for CACI at 3-months which reached significance at the 6-month follow-up (87% abstinence for CACI vs. 75, 63, and 75% for CATI, CA, and SC; *p* < .02). No group differences were found for illicit drug use at either 3 or 6 month follow-up.

**Conclusions:** No group differences were found on illicit drug use, with similar rates of use across the 2 intervention and 2 control groups, mirroring most other SBIRT trials. However, the computer-delivered intervention was associated with significantly less binge drinking at the six-month follow-up. Additional analyses will examine other outcome measures such as frequency (days) of use and urinalysis drug use assays.

## A67 Common factors and ethopoeia in computer-delivered brief intervention for alcohol use: a factorial trial

### Emily R. Grekin^1^, Jennifer D. Ellis^1^, Steven J. Ondersma^2^, Jessica R. Beatty^3^, Lucy McGoron^3^

#### ^1^Department of Psychology, Wayne State University, Detroit, MI, USA; ^2^Merrill Palmer Skillman Institute and Department of Psychiatry and Behavioral Neurosciences, Wayne State University, Detroit, MI, USA; ^3^Merrill Palmer Skillman Institute, Wayne State University, Detroit, MI, USA

##### **Correspondence:** Emily R. Grekin - grekine@wayne.edu

*Addiction Science & Clinical Practice* 2017, **12(Suppl 1)**: A67

**Background:** Media Equation Theory implies that common relationship factors, such as empathy and positive regard, are active in electronic brief interventions (e-BIs) just as they are in person-delivered interventions, particularly when lifelike characteristics are present. We will test this hypothesis by: (1) evaluating discriminability of common factors in an e-BI context; (2) measuring immediate post-e-BI changes in state motivation with and without common factors present; and (3) evaluating the effect of common factors, with and without the presence of lifelike characteristics, on changes in alcohol use at follow-up. Data from steps 1 and 2 are presented below; step 3 is ongoing as an NIAAA-funded factorial trial.

**Materials and methods:** In study 1, 22 participants viewed high empathy only, high positive regard only, and education-only content to evaluate discriminability. Pilot participants viewed the content for these cells in counterbalanced order and ranked them for preferability, understanding, and supportiveness. In study 2, 100 heavy-drinking undergraduates were randomly assigned to either a high- or a low-empathy e-BI. Intentions to reduce drinking were assessed both before and after the intervention, yielding a state motivation change score.

**Results:** In study 1, the high empathy e-BI was rated as most likeable (z = 3.01, *p* < .01), supportive (z = 3.01, *p* < .01), understanding (z = 3.47, *p* < .01), and affirming (z = 2.56, *p* = .01). Participant ratings for the control and positive regard conditions did not differ. In study 2, participants in the high-empathy condition reported greater increases in intentions to reduce drinking over the course of the study (β = .24, *p* < .05).

**Conclusions:** Results suggest that empathy within the context of an e-BI may increase acceptability and motivation to change. Findings are consistent with the Media Equation Theory and suggest that lifelike technology elicits social responses. The ongoing factorial trial (expected N = 352) will yield key data regarding differences in drinking at follow-up as a function of common factors, with and without a spoken voice or presence of an animated narrator.

